# Multimodal biomarker discovery for active *Onchocerca volvulus* infection

**DOI:** 10.1371/journal.pntd.0009999

**Published:** 2021-11-29

**Authors:** Ole Lagatie, Emmanuel Njumbe Ediage, Dirk Van Roosbroeck, Stijn Van Asten, Ann Verheyen, Linda Batsa Debrah, Alex Debrah, Maurice R. Odiere, Ruben T’Kindt, Emmie Dumont, Koen Sandra, Lieve Dillen, Tom Verhaeghe, Rob Vreeken, Filip Cuyckens, Lieven J. Stuyver

**Affiliations:** 1 J&J Global Public Health, Janssen R&D, Beerse, Belgium; 2 Discovery Sciences, Janssen R&D, Beerse, Belgium; 3 Department of Clinical Microbiology, School of Medicine and Dentistry, Kwame Nkrumah University of Science and Technology, Kumasi, Ghana; 4 Faculty of Allied Health Sciences, Kwame Nkrumah University of Science and Technology, Kumasi, Ghana; 5 Kenya Medical Research Institute, Centre for Global Health Research, Kisumu, Kenya; 6 Research Institute for Chromatography (RIC), Kortrijk, Belgium; National University of Ireland Galway, IRELAND

## Abstract

The neglected tropical disease onchocerciasis, or river blindness, is caused by infection with the filarial nematode *Onchocerca volvulus*. Current estimates indicate that 17 million people are infected worldwide, the majority of them living in Africa. Today there are no non-invasive tests available that can detect ongoing infection, and that can be used for effective monitoring of elimination programs. In addition, to enable pharmacodynamic studies with novel macrofilaricide drug candidates, surrogate endpoints and efficacy biomarkers are needed but are non-existent. We describe the use of a multimodal untargeted mass spectrometry-based approach (metabolomics and lipidomics) to identify onchocerciasis-associated metabolites in urine and plasma, and of specific lipid features in plasma of infected individuals (*O*. *volvulus* infected cases: 68 individuals with palpable nodules; lymphatic filariasis cases: 8 individuals; non-endemic controls: 20 individuals). This work resulted in the identification of elevated concentrations of the plasma metabolites inosine and hypoxanthine as biomarkers for filarial infection, and of the urine metabolite *cis*-cinnamoylglycine (CCG) as biomarker for *O*. *volvulus*. During the targeted validation study, metabolite-specific cutoffs were determined (inosine: 34.2 ng/ml; hypoxanthine: 1380 ng/ml; CCG: 29.7 ng/ml) and sensitivity and specificity profiles were established. Subsequent evaluation of these biomarkers in a non-endemic population from a different geographical region invalidated the urine metabolite CCG as biomarker for *O*. *volvulus*. The plasma metabolites inosine and hypoxanthine were confirmed as biomarkers for filarial infection. With the availability of targeted LC-MS procedures, the full potential of these 2 biomarkers in macrofilaricide clinical trials, MDA efficacy surveys, and epidemiological transmission studies can be investigated.

## Author summary

Today’s diagnosis of infection with the filarial parasite *Onchocerca volvulus* mainly depends on the microscopic analysis of skin biopsies and serological testing. The work presented here describes the use of multiple mass spectrometry-based screening methods (metabolomics and lipidomics) to search for biomarkers indicative of infection with *Onchocerca volvulus*. This resulted in the identification of elevated concentrations of the plasma metabolites inosine and hypoxanthine as biomarkers for filarial infection, and of the urine metabolite *cis*-cinnamoylglycine as biomarker for *O*. *volvulus*. Further evaluation of these biomarkers in a geographically distinct non-endemic population however invalidated the use of urine *cis*-cinnamoylglycine. These findings are of utmost importance as it not only opens new avenues in the development of non-invasive diagnostic tools for filarial infections, but also emphasizes the need for evaluation and validation of newly discovered biomarkers in different populations from different geographies.

## Introduction

Onchocerciasis, or river blindness, is an infectious disease caused by the filarial parasitic nematode *Onchocerca volvulus* with an estimated prevalence of current infection of 17 million people worldwide and 120 million people at risk. Although transmission occurs in the African Region, the Region of the Americas and the Eastern Mediterranean Region, 99% of infected people live in 31 African countries [1]. Life cycle stages of *O*. *volvulus* in the human host consist of adult worms called macrofilaria, and microfilaria. While the macrofilaria accumulate in subcutaneous onchocercomas, microfilaria migrate through the skin, eyes and other organs. Symptoms of the disease; rash, itching, skin lesions and eye lesions that ultimately can lead to blindness are the result of the host’s inflammatory response to dying microfilariae [2]. Treatment of onchocerciasis is mainly based on mass drug administration (MDA) through Community Directed Treatment with Ivermectin (CDTi) aimed at breaking the transmission cycle in affected communities [3,4]. Alternatively, the antibiotic doxycycline targets the bacterial endosymbiont *Wolbachia*, resulting in sterilization and to some extent also death of adult worms [5]. To be able to monitor and evaluate these MDA programs, epidemiological mapping is performed to identify all high-risk areas where ivermectin treatment is needed. These mappings are mainly based on examination of individuals for the presence of palpable onchocercomas, presence of microfilariae (mf) in skin biopsies, and also the rapid diagnostic test (RDT) for the detection of IgG4 antibodies to the parasitic antigen Ov16 [6–14]. However, the invasive nature of skin biopsies makes it increasingly unpopular while antibody-based tests have their limitations [15]. To improve the sensitivity of the existing serological tests, a combined test for Ov16 and OVOC3261 IgG4 detection was proposed [16]. Since repetitive annual ivermectin treatment is required to prevent further pathology caused by newly produced microfilariae, efforts have been undertaken to develop drugs with macrofilaricidal activity, directly targeting the adult worms [17–20]. There is a need for surrogate markers of infection and preferably of the presence of live and active adult worms [15].

Recently, a WHO report was made available describing the target product profile (TPP) to support preventive chemotherapy [21]. The minimal target analyte to be detected is an antigen or other biomarkers specific for live, adult female worms. A diagnostic clinical sensitivity of ≥60% was deemed sufficient, while a clinical specificity of ≥ 99.8% was found to be necessary. To overcome the shortcomings of the currently available diagnostic tools, a number of studies have been performed to identify metabolites in blood and urine that reflect infection status and possibly also intensity of infection [22–25]. The most promising metabolite that was discovered so far was the neurotransmitter derived N-acetyltyramine-O-glucuronide (NATOG) in urine [23,26–30]. Parasite-derived DNA or microRNAs have also been proposed as possible blood-based biomarkers for onchocerciasis, but were found to have limited utility [31–33]. Also new serological markers have been proposed, such as the peptide makers OvMP-1 and OvMP-23, and OvNMP-48 [34–38]. The use of these peptide markers was subsequently found to be limited (specificity significantly less than the required ≤99.8%), due to unexplained cross-reactivity in a population of school-age children in a non-endemic area in southwest Kenya [39]. In the work presented here, we conducted a study using both plasma and urine samples from nodule positive individuals that had very low or negative mf counts due to treatment with ivermectin. Mass spectrometric methods, specifically designed to detect a large range of small molecules, i.e. metabolites or lipids, were applied to allow untargeted identification of parasite derived molecules or host response markers. In a second phase, targeted liquid chromatography coupled to mass spectrometry (LC-MS) methods were developed and used to assess the concentrations of selected features in an extended validation sample set, leading to the confirmation of 3 candidate biomarkers, namely plasma hypoxanthine, plasma inosine and urine *cis*-cinnamoylglycine (CCG) [40]. The cinnamoylglycine candidate biomarker for onchocerciasis was more recently also identified by others [41]. In this study, we describe the full discovery process of these candidate biomarkers and evaluate the value of their performance considering the TPP from WHO in a sample set collected in a geographically distinct non-endemic region.

## Results

### Selection of sample sets for biomarker discovery and biomarker validation

We envisioned identifying biomarkers for *Onchocerca volvulus* infection and more in particular for the presence of macrofilaria. For all samples from Ghana (n = 253), an Ov16 RDT was performed. Based on this test, 68 of the 98 (69.4%) nodule positive individuals (NP), 26 of the 51 (51.0%) endemic controls (EC), 9 of the 54 (16.7%) non-endemic controls (NEC), and 12 of the 50 (24.0%) lymphatic filariasis patients (LF) were found to be seropositive. Given the high specificity reported for this test (97–98%), these data demonstrated that in the LF and NEC groups some onchocerciasis occurred, although at a lower prevalence than in the *O*. *volvulus* endemic population [8,9,42].

The discovery sample set consisted out of biomaterials of 68 nodule positive individuals that were Ov16 positive. The non-endemic control group consisted out of samples from 20 individuals that were Ov16 negative. The discovery set was further completed with 8 LF infected individuals that were Ov16 negative. An overview of the samples that were selected to be used in the discovery study is presented in S1 Table.

The validation sample set consisted out of the entire collection (n = 253) described above, complemented with biomaterials of 50 Belgian healthy controls.

### Biomarker discovery using untargeted approaches

All employed untargeted methodologies for comparative profiling of lipids (lipidomics) or metabolites (metabolomics) in plasma or urine, resulted in several features that met the pre-defined criteria (See S1 Supplementary Materials and Methods). All features detected using LC-MS were subjected to recursion analysis to remove false positives and a final list of features was prepared. For gas chromatography (GC)-MS analyses, only features that could be identified based on the available libraries were retained. Table 1 summarizes the number of features retained throughout the successive data-processing steps. The final lists of features that were retained as candidate biomarkers for onchocerciasis are presented in S2–S6 Tables.

**Table 1:** Number of features retained throughout the successive data-processing steps for the different methodologies employed. LC-MS based analyses were performed both in the positive and negative electrospray ionization mode (referred to as ESI+ and ESI-, respectively).

Data processing step	Feature number							
	Plasma					Urine		
	Lipidomics		Metabolomics			Metabolomics		
	ESI +	ESI -	ESI +	ESI -	GC-MS	ESI +	ESI -	GC-MS
Features extracted from full cohort	23,247	14,182	36,116	34,344	167	14,757	35,143	200
Features highly upregulated in nodule positives individuals	56	81	58	87	27	44	146	28
Upregulated features in NP shared with LF patients	37	28	42	57	2	27	93	1
Features retained after recursion (for LC-MS) or identification (for GC-MS)	19	21	14	35	22	5	25	16

**Fig 1:**
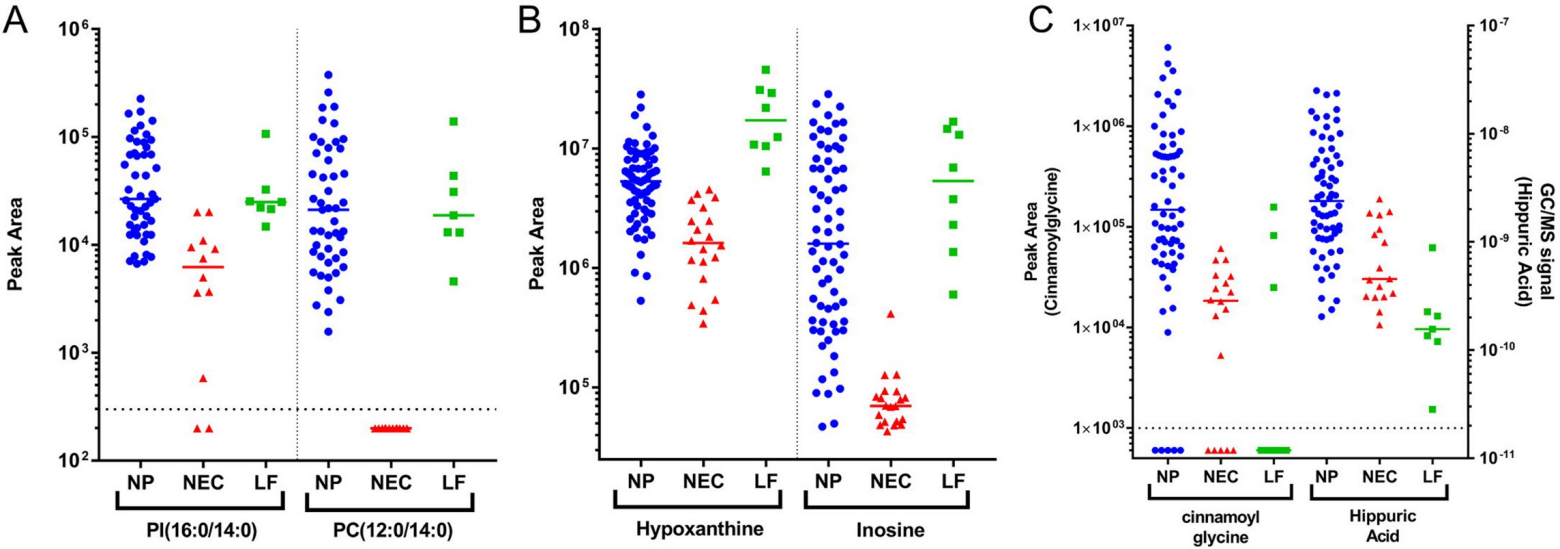
Selection of biomarkers associated to onchocerciasis. (*A*) Results for plasma lipids PI(16:0/14:0) and PC(12:0/14:0) in the different groups. *(B*) Results for plasma metabolites hypoxanthine and inosine in the different groups. (*C*) Results for urine metabolites cinnamoylglycine and hippuric acid in the different groups. All results are expressed in peak area. Abbreviations: NP: Nodule Positive; NEC: Non-Endemic Control; LF: Lymphatic Filariasis.

For each approach (urine metabolomics, plasma metabolomics and plasma lipidomics), two features were selected for further investigation:

- *Plasma PI(16*:*0/14*:*0) and PC(12*:*0/14*:*0)*
  In the lipidomics analysis, among the 34 features retained (S2 Table), the occurrence of several features with C12 and C14 fatty acid chains is notable. Two of them, namely phosphatidylinositol (PI)(16:0/14:0) and phosphatidylcholine (PC) (12:0/14:0) show more than 20-fold change between NEC and NP (Fig 1A). Clearly, these lipids are not specific for *O*. *volvulus* infection but rather reflect infection with a filarial helminth (both LF and onchocerciasis). Whereas PI(16:0/14:0) is markedly elevated in plasma from infected individuals, it is also present in lower quantities in the non-endemic controls. The occurrence of PC(12:0/14:0) in plasma however appears to be unique for individuals with filarial infection.
- *Plasma hypoxanthine* *and inosine*
  In the metabolomics analysis of the plasma samples, 71 features were retained (S3 and S5 Tables). Many of these have an unknown structure, but for several other features a structure could be derived from the MS/MS spectrum. Two of the most discriminating markers (based on fold change and statistical significance) could be identified as hypoxanthine and inosine. Both metabolites were elevated both in onchocerciasis and LF patients (Fig 1B). Hypoxanthine levels are significantly higher in LF patients compared to onchocerciasis (*P* = 0.0008). Inosine levels are similar in both patient populations (*P* = 0.06) but markedly higher than in in the NEC (*P*<0.0001 both for onchocerciasis and LF).
- *Urine cinnamoylglycine and hippuric* *acid*
  Upon analysis of the urine metabolome, a total of 46 features were retained for further analysis (S4 and S6 Tables). Besides several features with unknown molecular structure, also for urine many could be identified based on the MS/MS spectrum. Two of the most discriminating markers were identified as hippuric acid and cinnamoylglycine (Fig 1C). These molecules appear to be only elevated in urine from onchocerciasis patients and not from LF patients.

### Biomarker confirmation in discovery sample set using targeted approaches

Targeted LC-MS methods were developed for (i) PI(16:0/14:0) and PC(12:0/14:0) in plasma, (ii) for hypoxanthine and inosine in plasma, and (iii) for hippuric acid and cinnamoylglycine in urine. To proof the structure of the identified biomarkers and to be able to determine them quantitatively, synthetic reference material was obtained and used to develop the targeted LC-MS methods. The same plasma or urine sample extracts as used in the discovery study were re-analyzed using the targeted methods, and results of the comparison between both data sets are shown in Table 2 and S1 Fig. Also, receiver operating characteristic (ROC) analysis was performed on the data obtained using the targeted assays and Area Under Curve (AUC) values were calculated (S2 Fig). Only markers that could be considered good or excellent markers (AUC values above 0.80 or 0.90, respectively) were retained for further validation [43].

**Table 2:** Comparison between untargeted and targeted analysis of candidate biomarkers. Correlation curves were prepared on log-transformed data based on peak area from the untargeted analysis (X) and concentration (ng/mL) of the targeted analysis (Y) and correlation coefficients (r^2^) were calculated. Based on the data of the targeted analysis, ROC analysis was performed with NP and NEC as cases and controls, respectively and AUC was calculated.

	Equation	r^2^	AUC [NP vs NEC]
**Plasma markers**			
PI(16:0/14:0)	Y = 0.7172*X - 1.953	0.4813	0.7720
PC(12:0/14:0)	Y = 0.7968*X - 2.976	0.5653	0.7148
Hypoxanthine	Y = 0.9996*X - 3.027	0.9263	0.8612
Inosine	Y = 1.165*X - 5.292	0.9622	0.9465
**Urine markers**			
Hippuric acid	Y = 0.1184*X + 7.645	0.05429	0.7022
*Trans*-cinnamoylglycine	Y = 0.3664*X + 3.178	0.4226	0.7222
*Cis*-cinnamoylglycine	Y = 0.7487*X + 0.1196	0.854	0.9034

- *Plasma PI(16*:*0/14*:*0) and PC(12*:*0/14*:*0)*
  For the plasma lipids PI(16:0/14:0) and PC(12:0/14:0), a moderate correlation was obtained between both data sets (r^2^ = 0.48 and 0.57, respectively) resulting also in markedly reduced AUC in the ROC analysis (AUC = 0.77 and 0.71, respectively). Although generally the same trend as in the discovery data set is still apparent, based on the AUC values, these markers could be considered only fair markers (AUC 0.70–0.80). It was therefore decided not to further explore both lipids as markers for active *O*. *volvulus* infection.
- *Plasma hypoxanthine* *and inosine*
  For the plasma metabolites hypoxanthine and inosine correlation between both data sets were within preset acceptance criteria (r^2^ = 0.93 and 0.96, respectively), confirming the identity of these molecules and validating the use of the targeted LC-MS method. With AUC values of 0.86 and 0.95, for hypoxanthine and inosine respectively, both features were considered of interest for further evaluation.
- *Urine cinnamoylglycine* *and hippuric acid*
  For the urine metabolites, the data of hippuric acid could not be confirmed using the targeted method (r^2^ = 0.05). Cinnamoylglycine occurs both as *cis*- and *trans*-isomer and since it could not be deduced from the discovery experiments which isomer was identified, both synthetic molecules were included. Targeted LC-MS analysis demonstrated that the *cis*-isomer was the urinary biomarker for onchocerciasis, as the data obtained for the *cis*-isomer were concordant with the discovery data (r^2^ = 0.85) while for the *trans*-isomer this was weak (r^2^ = 0.42). For the *cis*-isomer, eight samples that were undetectable in the untargeted method, now had low but detectable levels of *cis*-cinnamoylglycine (CCG). This discrepancy might be due to a difference in sensitivity of the targeted method compared to the untargeted method. Exclusion of these discrepant samples would result in a r^2^ of 0.96, re-confirming the identity of the discovered feature to be the *cis*-isomer of cinnamoylglycine and warranting its further evaluation in the validation set. ROC analysis of the targeted analysis data re-confirmed the diagnostic potential of CCG with an AUC of 0.90.

In conclusion, the conversion of the biomarker features from the untargeted approach into confirmed features using a targeted LC-MS approach was successful for CCG, inosine, and hypoxanthine. The PI(16:0/14:0), PC(12:0/14:0), and hippuric acid biomarker features did not meet the preset acceptance criteria, and were hence not further evaluated in the validation set.

### Biomarker validation in validation sample set

**Fig 2:**
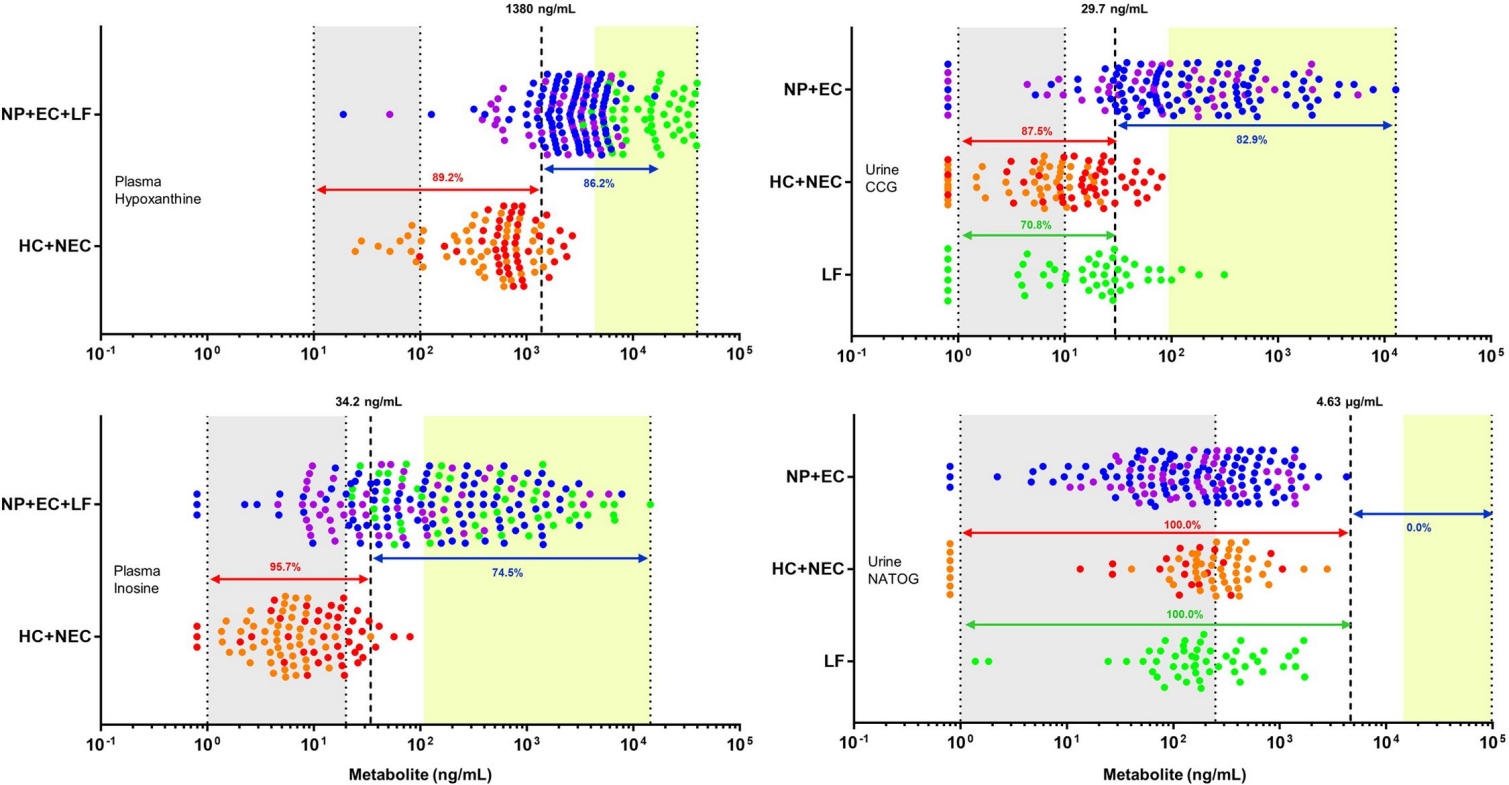
Validation of biomarkers associated to onchocerciasis. Results for the three new biomarkers (plasma hypoxanthine and inosine; and urine CCG) and for NATOG that have been obtained on the nodule positive individuals (NP, blue), endemic controls (EC, purple), LF patients (LF, green), non-endemic controls (NEC, red) and healthy controls from Belgium (HC, orange). For each plot, the grey zone indicates the zone between limit of detection (LOD) and limit of quantification (LOQ), the dashed line indicates the biomarker-specific cutoff and the yellow zone indicates the zone between 0.5 log times the cut-off and the maximum value observed for the specific biomarker. In case of NATOG, the cutoff (4.63 μg/mL = 13 μM) and maximum value (98.3 μg/mL = 276 μM) was derived from the data published by Globisch and colleagues [27]. NATOG data were previously obtained on the same sample set (doi: 10.1186/s13071-016-1582-6) [28]. For each marker, the percentage of positive samples in the group considered to be infected (i.e. sensitivity) and the percentage of negative samples in the group considered to be not infected (i.e. specificity), is indicated. For CCG and NATOG, which are onchocerciasis specific biomarkers, the LF group was plotted separately from the other control samples to highlight specificity.

The levels of hypoxanthine and inosine in plasma, and of CCG in urine were further evaluated in the validation sample set (Fig 2). Based on these data, ROC analysis was performed, biomarker specific cutoffs were defined, and diagnostic characteristics were determined (Table 3). Since the discovery sample set confirmed that both plasma biomarkers hypoxanthine and inosine are elevated in plasma of nodule positive individuals as well as in LF infected individuals, ROC analysis for these markers was performed using the Belgian healthy controls and the non-endemic controls as negative panel and the nodule positives, LF infected individuals and endemic controls as positive panel. The urine biomarker CCG appeared to be specifically elevated in the onchocerciasis group and not in the LF group, based on the discovery sample set, and therefore ROC analysis was performed using the Belgian healthy controls, the non-endemic controls and LF infected individuals as negative panel and the nodule positives and endemic controls as positive panel (S3 Fig).

**Table 3:** Diagnostic characteristics of the biomarkers, as determined on the validation sample set. Sensitivity for filarial markers was based on NP, LF and EC, while specificity was based on NEC and HC. For onchocerciasis markers, sensitivity was based on NP and EC, with specificity based on NEC, HC and LF.

	AUROC	Cutoff (ng/mL)	Sensitivity (%)	Specificity (%)	NP positive (%)	LF positive (%)	EC positive (%)	HC positive (%)	NEC positive (%)
**Plasma filariasis markers**									
Number of samples included in each group (n)					95	50	51	49	53
Hypoxanthine	0.93	1380	86.2%	89.2%	85.3%	100.0%	74.5%	4.1%	18.9%
Inosine	0.91	34.2	74.5%	95.7%	74.7%	94.0%	52.9%	0.0%	9.4%
**Urine onchocerciasis markers**									
Number of samples included in each group (n)					96	48	50	44	52
*Cis*-cinnamoylglycine	0.87	29.7	82.9%	82.2%	88.5%	29.2%	72.0%	0.0%	23.1%

Abbreviations: NP: nodule positive; LF: Lymphatic Filariasis; EC: Endemic Control; HC: Healthy Control; NEC: Non-Endemic Control

- *Plasma* *hypoxanthine and inosine*
  Based on the ROC analysis, cutoffs for plasma hypoxanthine and inosine were set at 1380 ng/mL, and 34.2 ng/mL, respectively. Based on these cutoffs, hypoxanthine had a sensitivity of 86.2% and a specificity of 89.2%, while for inosine sensitivity was 74.5% and specificity was 95.7%. The obtained quantitative results confirm our initial observation that both hypoxanthine and inosine are elevated in plasma of nodule positive individuals, endemic controls as well as in LF patients compared to non-endemic controls (P<0.0001). These data also confirm the more pronounced elevation of hypoxanthine and inosine in LF patients compared to onchocerciasis patients (P<0.0001). Based on hypoxanthine levels, no difference could be observed between nodule positive individuals and endemic controls (P = 0.2578). For inosine, levels are significantly lower in the endemic control group (P = 0.0010), but with a large overlap between both groups.
- *Urine* *CCG*
  The data obtained for urinary CCG in the validation sample set reinforce the potential of this urinary metabolite as a biomarker for onchocerciasis. Based on the ROC analysis, a cutoff was defined at 29.7 ng/mL, resulting in 82.9% sensitivity and 82.2% specificity. The NEC samples had levels that were higher than the healthy control samples (P<0.0001), with 12 of the 52 non-endemic control samples above the cutoff, hence considered false positive. This observation might need further evaluation. Also in the LF group, 14 out of 48 samples appeared to be positive for urinary CCG. None of the healthy control samples were positive for CCG. Similar to plasma inosine, CCG levels are largely overlapping in the nodule positive and endemic control groups, with on average lower concentrations in the endemic control group (P = 0.0449). When assessing both groups separately, 88.5% of the nodule positive individuals was positive while for the endemic control group this was only 72.0%.

### Evaluation of the biomarkers in a non-endemic population from Kenya

**Fig 3:**
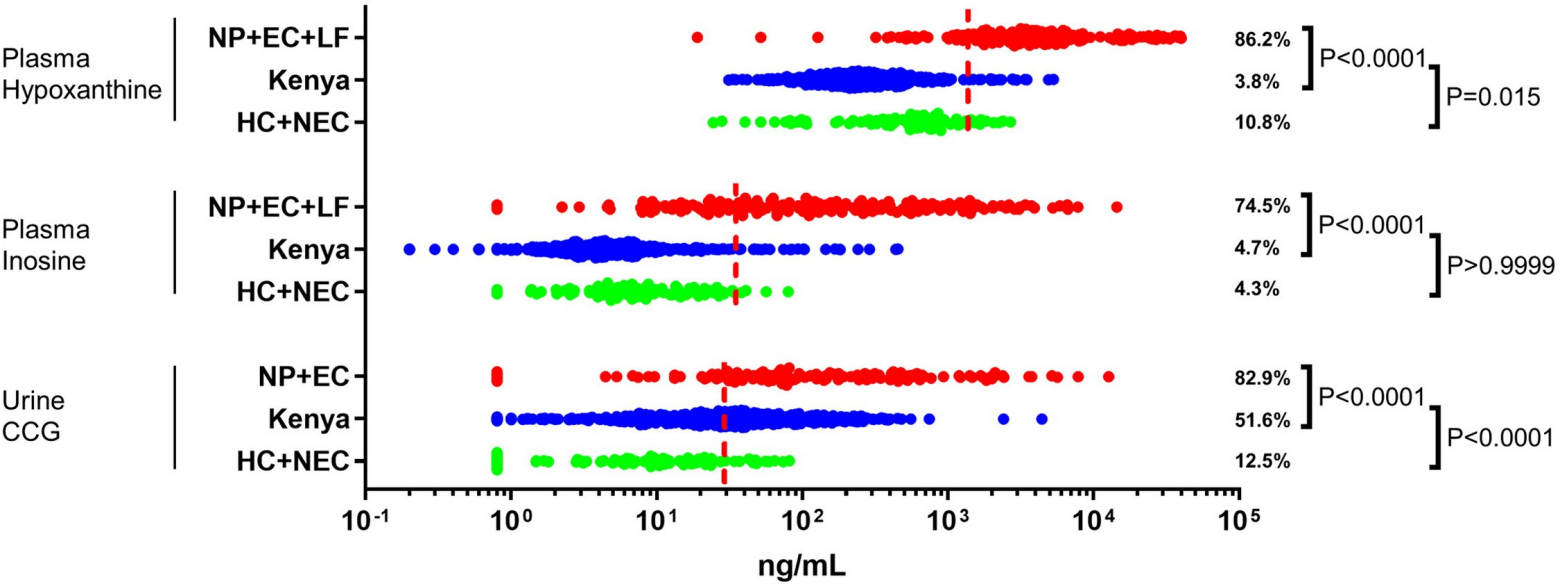
Levels of plasma hypoxanthine, plasma inosine, and urine CCG in a non-endemic population from Kenya, compared to the positive and negative population from the validation study. The dashed lines indicate the biomarker-specific cutoffs. The percentage of positive samples in each group is indicated as well as P-value of Chi square analysis for each metabolite comparing the Kenyan population with the negative and positive validation set, respectively.

**Fig 4:**
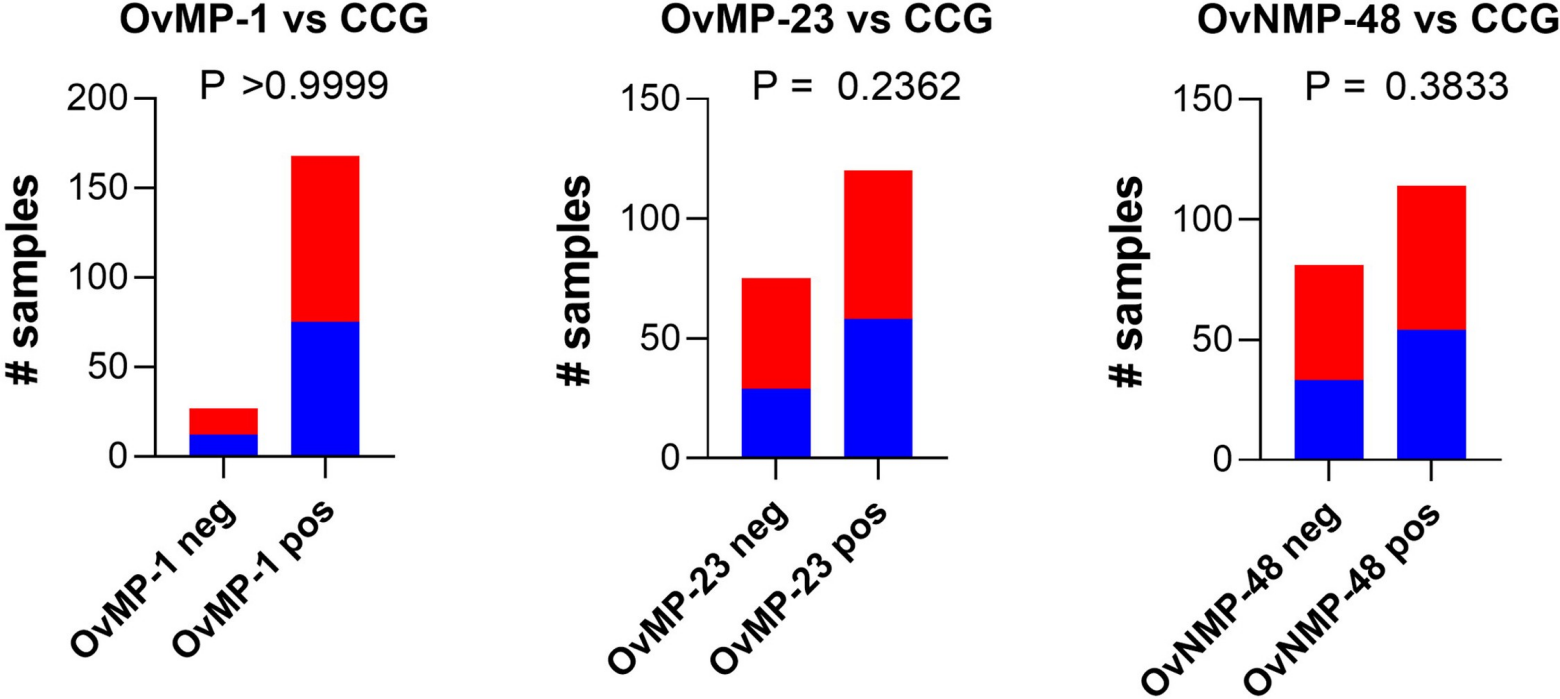
The percentage of individuals that were urine CCG positive stratified according to their peptide serology status OvMP-1, OvMP-23 and OvNMP-48 [39]. Red bars indicate number of samples positive for urine CCG, blue bars indicate number of samples negative for urine CCG. For each peptide the P-value of Chi square analysis is indicated.

In order to evaluate the specificity of the new biomarkers, the levels of hypoxanthine and inosine in plasma, and of CCG in urine were evaluated in a sample set collected in the southwest part of Kenya (Fig 3). Kenya is non-endemic for *O*. *volvulus* and lymphatic filariasis is mainly confined to the coastal region, which is different from the region where this sample set has been collected [44–47]. Out of 476 study participants, 3.8% and 4.7% were found to be positive for plasma hypoxanthine and inosine, respectively. Also, Chi square analysis indicated that the Kenyan population was indistinguishable from the negative validation set based on plasma inosine levels (P > 0.9999). Based on plasma hypoxanthine levels, there was a weak statistical difference with the negative validation set (P = 0.015) but this appears to be caused by the rather high number of false positives in the validation set. These data confirm the biomarker potential for plasma hypoxanthine and inosine as filarial markers. On the other hand, 51.6% of the Kenyan participants were found to be positive for CCG in urine. The CCG levels in this non-endemic population overlapped almost entirely with the levels detected in both the positive and negative sample set from the validation study. This observation suggests that urine CCG is not a good biomarker for onchocerciasis. Since many of the study participants were infected with soil-transmitted helminths or *Schistosoma mansoni*, we grouped samples based on these infections. No significant difference between the non-infected and different infection groups was observed (P_χ_^2^ = 0.7042), suggesting that none of these intestinal parasites are linked to elevated CCG levels. The urine CCG data were also compared with the peptide serology data previously obtained from the same study population [39]. None of the 3 peptide markers correlated with urine CCG, with P-values from Chi square analysis ranging from 0.2362 to >0.999 (Fig 4). This observation indicated that the elevated excretion of CCG and positive peptide serostatus in this population are not caused by the same study- or region-specific factor.

## Discussion

Onchocerciasis remains an important health issue in several African countries, despite the MDA programs that have been put in place. To better steer these programs, novel tools for epidemiological mapping are urgently needed, besides skin biopsies and antibody-based tests, with specifications as presented in the TPP from WHO [21]. Also, for the development of macrofilaricide drugs, good pharmacodynamic markers will be required to monitor the effect of these drug candidates on the adult worms. In the work presented here, we have used urine and plasma metabolomics and plasma lipidomics approaches to identify novel molecules that have potential as diagnostic markers.

In plasma, 2 molecules were identified with promising diagnostic characteristics: the metabolites hypoxanthine and inosine. Both plasma markers were found to be non-specific for onchocerciasis but were rather indicative of a filarial infection. With a sensitivity of 86.2% and 74.5%, and a specificity of 89.2% and 95.7%, respectively for hypoxanthine and inosine, both markers might warrant further research into the clinical utility as filarial markers. For LF specifically, the data indicate that hypoxanthine is a stronger biomarker than inosine as hypoxanthine had a 100% sensitivity for detecting LF, while for inosine only 94.0% of samples had levels above the cutoff. Both metabolites are products of the purine degradation pathway, with inosine the first step in the catabolism of adenosine, which is then being further degraded into hypoxanthine [48,49]. In man, this is then further metabolized into uric acid by the enzyme xanthine oxidase, and subsequently excreted in urine [50]. Helminths however lack this enzyme and consequently have hypoxanthine as end product of their purine metabolism [51]. Both molecules were also identified as being significantly upregulated in a metabolite profiling study that was performed on plasma samples from microfilaridermic patients (> 50 mf/mg skin) [24]. The fact that also in our study population with no or very low levels of mf in the skin, a similar increase is observed, might be indicative that the adult worm is (partly) responsible for the accumulation of both metabolites. Why plasma hypoxanthine levels–and to some extent also plasma inosine levels–in LF patients are even further increased is not clear based on the currently available data, but it is possible that differences in infection intensity play a role.

In urine from individuals residing in an endemic area, CCG was found to be specifically upregulated in onchocerciasis patients and not in LF patients, with a sensitivity of 82.9% and a specificity of 82.2%. Cinnamoylglycine is one of the metabolites that is produced upon degradation of cinnamic acid or one of its derivatives, such as e.g. caffeic acid and ferulic acid [52]. Whereas its most abundant metabolite, hippuric acid, does not form stereoisomers, the minor metabolite cinnamoylglycine can occur in both *trans*- and *cis*-configuration. Since most cinnamic acid present in nature is *trans*-cinnamic acid, this will also give rise to *trans*-cinnamoylglycine [53]. We found indeed that in the western healthy control population, all (100%) urine samples contained very low levels of CCG, with maximal level detected at 21.6 ng/mL, which is still substantially lower than the cutoff that was set at 29.7 ng/mL. In the onchocerciasis endemic population that was investigated, 82.9% of all urine samples contained levels above this cutoff. In the group of nodule positive individuals specifically, this was even 88.5%. However, investigation of a non-endemic population from Kenya, a country declared free of onchocerciasis [44], demonstrated that urine CCG was also detected at similarly high levels in more than half of the tested individuals, suggesting that CCG excretion in urine of individuals in Kenya is not related to *O*. *volvulus* infection.

We have previously reported on the discovery and value of these biomarkers in *Onchocerca* endemic areas [40]. In a more recent publication, Wewer *et al*. also identified cinnamoylglycine as a candidate biomarker for onchocerciasis in endemic areas [41]. Although not further investigated and hence not confirmed, it is very well possible that the marker they identified is in fact also *cis*-cinnamoylglycine. It is difficult to compare the data from both studies as no quantitative method has been used to determine cinnamoylglycine in the Wewer *et al*. study. However, the authors observed that the difference between individuals with onchocerciasis and non-infected controls was not significant and that only 17.2% of the onchocerciasis group had urine cinnamoylglycine levels higher than the highest value of the control samples. Taken together, the authors concluded that cinnamoylglycine is suitable to identify infected individuals with very high metabolite levels, but with a large variation. Our results add a further restriction to that observation, namely that the CCG cannot be considered as a diagnostic marker for onchocerciasis when outside of the endemic area. Given the WHO TPP requirements on specificity, CCG should not be considered as a useful contribution to the *Onchocerca* biomarker armamentarium.

The levels of CCG might be influenced by specific dietary patterns in the study populations investigated here, as cinnamic acid is a molecule that is widely present in plants. The *cis*-isomer of cinnamic acid is produced in plants by photoisomerization of *trans*-cinnamic acid but is typically detected only in trace amounts in plants [54,55]. It is possible that there are substantial regional differences in baseline CCG levels, caused by different nutritional or living habits. Since the cutoff for CCG positivity was only based on samples collected in Ghana, this might explain the high number of CCG positive individuals in Kenya. It would however mean that specific cutoffs need to be determined for different countries or regions, making it practically very hard to use. Next to the possible dietary effect, other helminth infections might play a role, but based on the data obtained in this study it appears that soil-transmitted helminthiasis and schistosomiasis are not linked to the increased excretion of CCG.

A test for a metabolite biomarker for onchocerciasis such as urinary CCG could have been a promising tool to identify those individuals that are currently missed based on clinical examination. However, the work here shows the importance of demonstrating clinical utility in biomarker research. Biomarker discovery studies, even when well-executed with a proper test and validation set, are typically based on sample sets from one specific origin. Especially in the context of tropical diseases, it is not always easy to have proper control groups. Ideally, these should be as similar as possible to the infected group, but only differing in their infection status. Often—also in this study—a negative control group is obtained from a city in the vicinity of the endemic region. However, these individuals do not only differ in their infection status, but also in their diet, specific exposures to other pathogens, occupation… Confirmation studies in geographically different populations are absolutely essential to ensure that the right biomarkers are being selected for further development into diagnostic tools.

In our previous work on clinical utility testing of peptide biomarkers, we came to a similar observation when investigating this population of children in Kenya [39]. More than 50% of the children were indeed seroreactive to the peptide epitopes, without presenting any evidence for being infected, or being exposed, or residing in an endemic area. This observation invalidated the peptide biomarker concept as an additional tool. In this study, the same population of children also showed elevated levels of CCG, again without any evidence of *O*. *volvulus* exposure or infection. It should also be noted that there is no correlation between the signals observed on the peptide serology and elevated CCG levels. Both are independent observations and again emphasize the need for confirmation studies.

To be useful as a pharmacodynamic (PD) marker to monitor the efficacy of new drugs in clinical trials, it is important that a biomarker covers a sufficiently large dynamic range in the study population. We reasoned that a metabolite would need to be present at least at a concentration 0.5 log higher than the defined cutoff to allow proper pharmacodynamic modeling upon treatment. This permits detailed longitudinal monitoring of the drug’s effect on the disease and worm activity. Based on the data for the candidate biomarkers described here, we suggest plasma inosine as PD marker for onchocerciasis treatment (Fig 2) as for this marker, a sufficiently high number of infected individuals are found in the window above this PD cutoff, which is not the case for hypoxanthine. To confirm their use as PD marker, retrospective analysis of previously executed (animal) studies and prospective collection of samples from macrofilaricide treated individuals will be required. Both the *O*. *ochengi* cow model and the SCID mouse *O*. *ochengi* implant model might be ideally suited to follow the increase of the suggested biomarkers under controlled experimental conditions and eventually also the decrease upon macrofilaricide treatment [56,57]. Also retrospective analysis of samples from doxycycline field studies might be useful to study the biomarker levels as it’s been described that doxycycline has macrofilaricide properties [18, 58].

Previous studies setup to discover biomarkers for onchocerciasis have identified NATOG as a urinary biomarker for onchocerciasis [23,26,27]. We included the NATOG data that were previously obtained on the same sample set in this work in Fig 2 [28]. This allows proper comparison of the newly identified biomarkers with NATOG. As was already described before, no increase in urinary NATOG levels were detected in the onchocerciasis group, with no samples having NATOG levels above the 13 μM cutoff (i.e. 4.63 μg/mL) that was defined by Globisch and colleagues [27]. Furthermore, no difference was observed with the control groups (non-endemic controls and healthy controls). It’s important to emphasize that the samples used in this study were collected from individuals with palpable nodules but without or with very low levels of microfilaria in the skin. The data for NATOG, in contrast to those obtained for plasma inosine, might suggest that NATOG should be considered a surrogate marker for the presence of microfilaria, rather than a surrogate for the presence of live adult worms.

In conclusion, this work shows the potential of plasma inosine and hypoxanthine as markers for filarial infection. Furthermore, plasma inosine shows potential to be used as pharmacodynamic marker for use in clinical trials investigating the efficacy of filaricides for treatment of onchocerciasis or lymphatic filariasis.

## Methods

### Ethics statement

Field study performed in Ghana was approved by the Committee on Human Research, Publications and Ethics of the School of Medical Sciences of the Kwame Nkrumah University of Science and Technology, Kumasi, Ghana and all study subjects signed an informed consent form. Plasma and urine samples from Kenya were collected as part of a field study. The study was approved by the KEMRI Scientific and Ethics Review Unit (SERU), Nairobi, Kenya (Protocol Nr. # KEMRI/SERU/CGHR/102/3554). Since all study participants were minors, informed consent forms were signed by parents/guardians of the study participants, and verbal assents were obtained from all study participants. Collection of samples from healthy donors in Belgium was approved by The Ethics Committee [“Commissie voor Medische Ethiek—ZiekenhuisNetwerk Antwerpen (ZNA) and the Ethics Committee University Hospital Antwerp] and an Informed consent was signed by all subjects. All samples used in this study were anonymized and were collected from adults (18 years or above) only.

### Study samples

Plasma and urine samples used for biomarker discovery were collected as part of a field study in Ghana as described before [28]. A total of 98 nodule positive subjects that donated plasma and urine samples were included, as well as 51 endemic controls that had no visible signs of onchocerciasis. Additionally, plasma and urine of samples from 54 non-endemic controls (from Kumasi, Ashanti Region) and 50 lymphatic filariasis patients (from Ahanta West District, Western Region) were available for testing. As an additional control group, plasma and urine samples from 50 Belgian healthy controls were included [59–63]. Samples for the biomarker evaluation study were collected in the former Nyanza province, in the southwest part of Kenya, with collections in the Kisumu county (high *S*. *mansoni* prevalence area) and Siaya county (high STH prevalence area). Parasitological information for this study sample set has been published before [64,65]. Stool samples were collected in order to determine the STH and *S*. *mansoni* infection status of these study participants. An overview of all study populations, including microfilarial (mf) load in the skin and mass drug administration information is provided in S7 Table. All blood and urine samples were stored in cold boxes before being processed in the lab. The plasma and urine samples were then stored at -80°C until analysis.

### Onchocerciasis IgG4 rapid test

The presence of IgG4 antibodies against the *O*. *volvulus* antigen Ov16 was determined using the SD BIOLINE Onchocerciasis IgG4 test (Standard Diagnostics, Gyeonggi-do, Republic of Korea), according to manufacturer’s instructions. Briefly, 10 μL of plasma was added to the round sample well on the lateral flow strip, immediately followed by the addition of 4 drops of assay diluent into the square assay diluent well. After 1 hour, tests were scored. Tests were considered positive only when both the test and control line were visible. Faint lines were considered positive, as recommended by the manufacturer.

### Preparation of QC samples

A quality control (QC) pool was constructed by collecting 50 or 100 μL of all the plasma or urine samples, respectively, that were used for the untargeted discovery approaches. Subsequently, this QC pool was divided into aliquots to acquire representative QC samples. QC samples were prepared simultaneously along with study samples and were analyzed throughout the LC-MS and GC-MS analysis sequences every five study samples. Since these samples do not contain any biological variability, they can be considered as technical replicates. For both plasma and urine, study and QC samples were prepared in random order. Blank extracts were prepared simultaneously along with study samples and were analyzed before the LC-MS and GC-MS analysis sequences to check the overall contamination in the analytical pipeline.

### Sample preparation and analysis

All sample preparation procedures, as well as all sample analyses (both untargeted and targeted approaches) are described in S1 Supplementary Materials and Methods [66–68]. All reference materials used in the targeted analysis were purchased from commercial suppliers, except for *cis-*cinnamoylglycine which was synthesized in-house. A detailed description of the synthesis and quality control procedures is available in S1 Supplementary Materials and Methods.

### Quality of analysis of the untargeted approaches

To monitor stability of the data during the analytical sequence, the total lipid or metabolite intensity of the QC samples is monitored in function of analysis time. For the metabolomics studies (both LC-MS and GC-MS), stable trends were observed for all sequences. For the lipidomics studies, an intensity drop was observed after QC sample 6. Therefore, all samples analyzed before this QC sample, were ruled out for further data processing, leaving only 49 NP, 12 NEC and 7 LF study samples for the comparative lipidomics study.

The validity of the performed analyses was monitored in both a targeted and a non-targeted manner using the QC samples. For the LC-MS based metabolomics and lipidomics, targeted monitoring was performed by determining the error of the measurement on signal intensity (peak area), retention time and mass accuracy for a list of 18–22 randomly selected metabolites. S8 Table summarizes the results of this targeted validity verification. Peak area fluctuations, originating from both the sample preparation step and the LC-MS analysis, are typically below 15% relative standard deviation, except for lipidomics, where these are typically below 30% relative standard deviation because of the more complex extraction procedure [69,70]. Chromatographic retention time reproducibility is in general satisfactory and less than 1 RSD%. Also, high mass accuracy (< 5ppm) was obtained for all analyses. For GC-MS based metabolomics, a normalization strategy was employed on all detected features. For plasma, next QC normalization was employed for all features. For urine, normalization for the Total Metabolite Content and Internal Standard was employed to compensate for the inherent dilutional differences between urine samples. Precision on peak area was calculated for a randomly selected range of identified metabolite species measured in the QC samples. S9 and S10 Tables summarize the results of this targeted validity verification. Peak area fluctuations are typically below 15% relative standard deviation for plasma and below 30% for urine.

Apart from this targeted approach, the reproducibility of the applied metabolomics analysis was examined in a more comprehensive way by calculating the error on all detected features in the QC samples and representing the acquired RSD distribution as depicted in S4 Fig. For all analyses performed, > 75% of all features had an RSD below 30%, which can be defined as the upper limit for untargeted or discovery metabolomics analysis [71]. For lipidomics in positive ESI however, only 58% of all features had an RSD below 30%, which can be explained by the large number of MS saturated lipids in positive ESI mode. The high lipid load was deliberately chosen for the detection of lower abundant lipid markers. For GC-MS, the data confirm the requirement for proper normalization procedures.

### Statistical analysis

Statistical analyses used to analyze the data obtained in the untargeted discovery studies, have been described in S1 Supplementary Materials and Methods. For evaluation of the correlation between data obtained using the untargeted approach and the targeted approach, linear regression analysis was performed on log-transformed data. A minimal r^2^ of 0.9 was set as acceptance criterion to justify the use of the targeted method for subsequent validation studies. For comparison of different groups in the validation studies, two-tailed unpaired t-test with Welch’s correction on log-transformed data was performed. ROC analysis was performed using specified sample sets as cases and controls, and cutoffs were determined as the point with maximal Youden’s index ((Sensitivity + Specificity)-1). Based on these cutoffs, sensitivity and specificity of each biomarker was determined, as well as percentage positives in specific sample sets. To determine whether more samples were found to be positive in one group compared to another group, contingency tables were prepared, and Chi square test was performed. All analyses were performed using GraphPad Prism version 7.00.

## Supporting information

S1 Fig Correlation between data obtained in the untargeted -omics approach vs. data obtained using targeted method. NEC samples have been indicated in red, LF samples in green and NP samples in blue.
(TIFF)

S2 Fig ROC analysis of the markers that were further investigated in a targeted LC-MS/MS analysis. ROC analysis was performed with NP and NEC as cases and controls, respectively.
(TIFF)

S3 Fig ROC analysis of the filarial markers hypoxanthine and inosine and the onchocerciasis marker CCG based on the data obtained from the validation sample set. Cutoff defined by maximal Youden’s index is indicated in red.
(TIFF)

S4 Fig RSD distribution on all detected features in the QC samples. (TIFF)

S1 Table Overview of samples used in metabolomics and lipidomics discovery study. (DOCX)

S2 Table Characteristics of features selected from the comparative plasma lipid profiling study. (DOCX)

S3 Table Characteristics of features selected from the comparative LC-MS based plasma metabolite profiling study. (DOCX)

S4 Table Characteristics of features selected from the comparative LC-MS based urine metabolite profiling study. (DOCX)

S5 Table Characteristics of features selected from the comparative GC-MS based plasma metabolite profiling study. (DOCX)

S6 Table Characteristics of features selected from the comparative GC-MS based urine metabolite profiling study. (DOCX)

S7 Table Overview of study population. (DOCX)

S8 Table Targeted validity verification of LC-MS based metabolomics and lipidomics. (DOCX)

S9 Table Targeted validity verification of GC-MS based metabolomics in plasma. Precision obtained with different normalization strategies for the QC samples is shown.
(DOCX)

S10 Table Targeted validity verification of GC-MS based metabolomics in urine. Precision obtained with different normalization strategies for the QC samples is shown.
(DOCX)

S1 Supplementary Materials and Methods Sample preparation, analytical procedures and synthesis of cis-cinnamoylglycine. (DOCX)
