# Supplementary figures and images for: Multimodal biomarker discovery for active Onchocerca volvulus infection
Source: PLoS Negl Trop Dis. 2021 Nov 29;15(11):e0009999. doi: 10.1371/journal.pntd.0009999 (PMC8659328; doi:10.1371/journal.pntd.0009999)

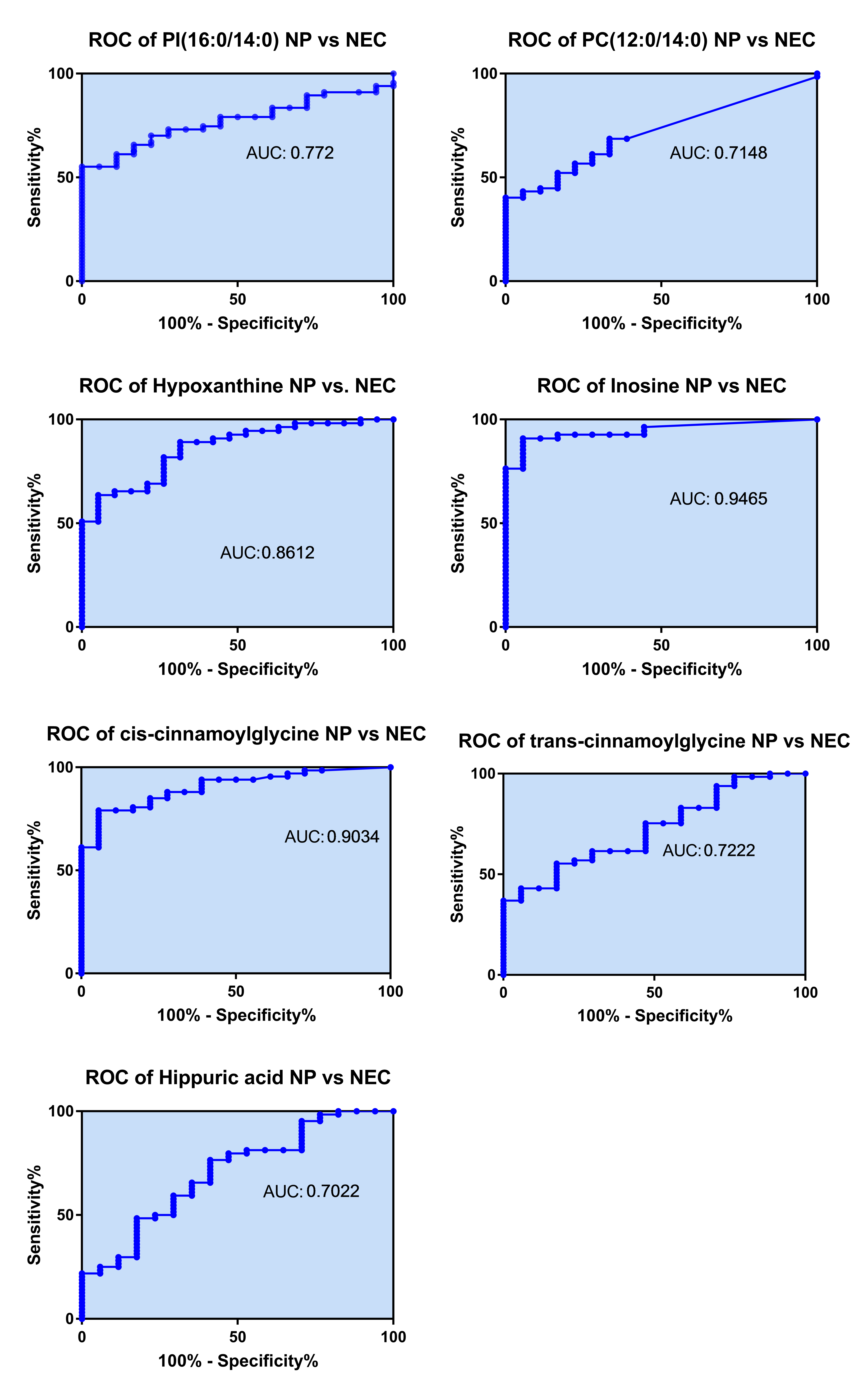

Supplement: S2 Fig — ROC analysis was performed with NP and NEC as cases and controls, respectively. (TIFF) [file pntd.0009999.s002.tiff]

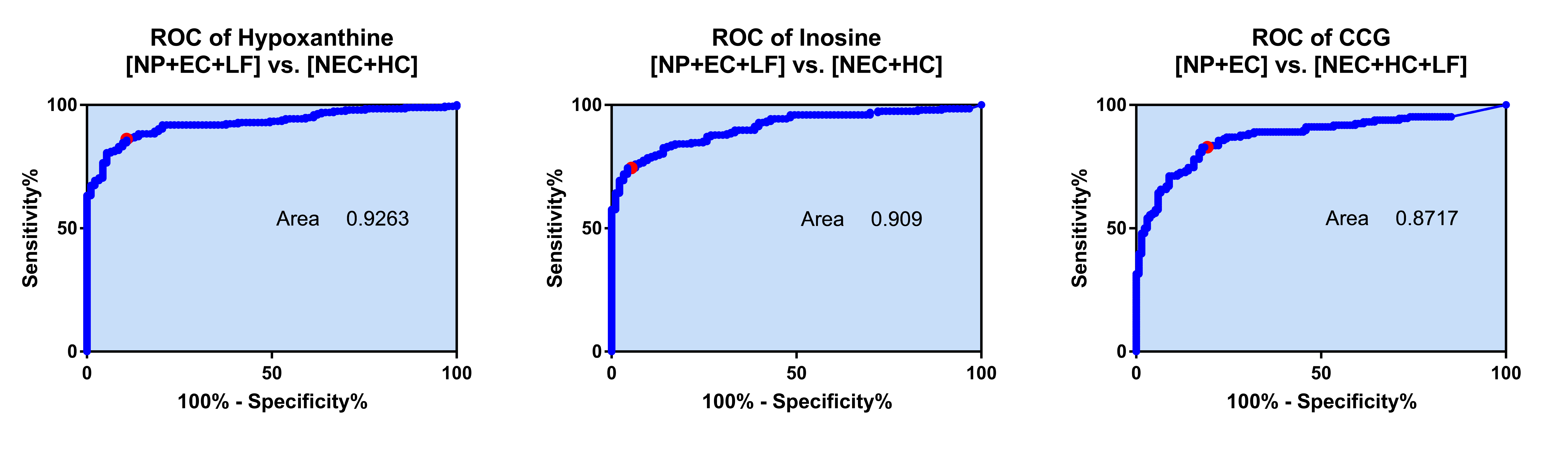

Supplement: S3 Fig — Cutoff defined by maximal Youden’s index is indicated in red. (TIFF) [file pntd.0009999.s003.tiff]

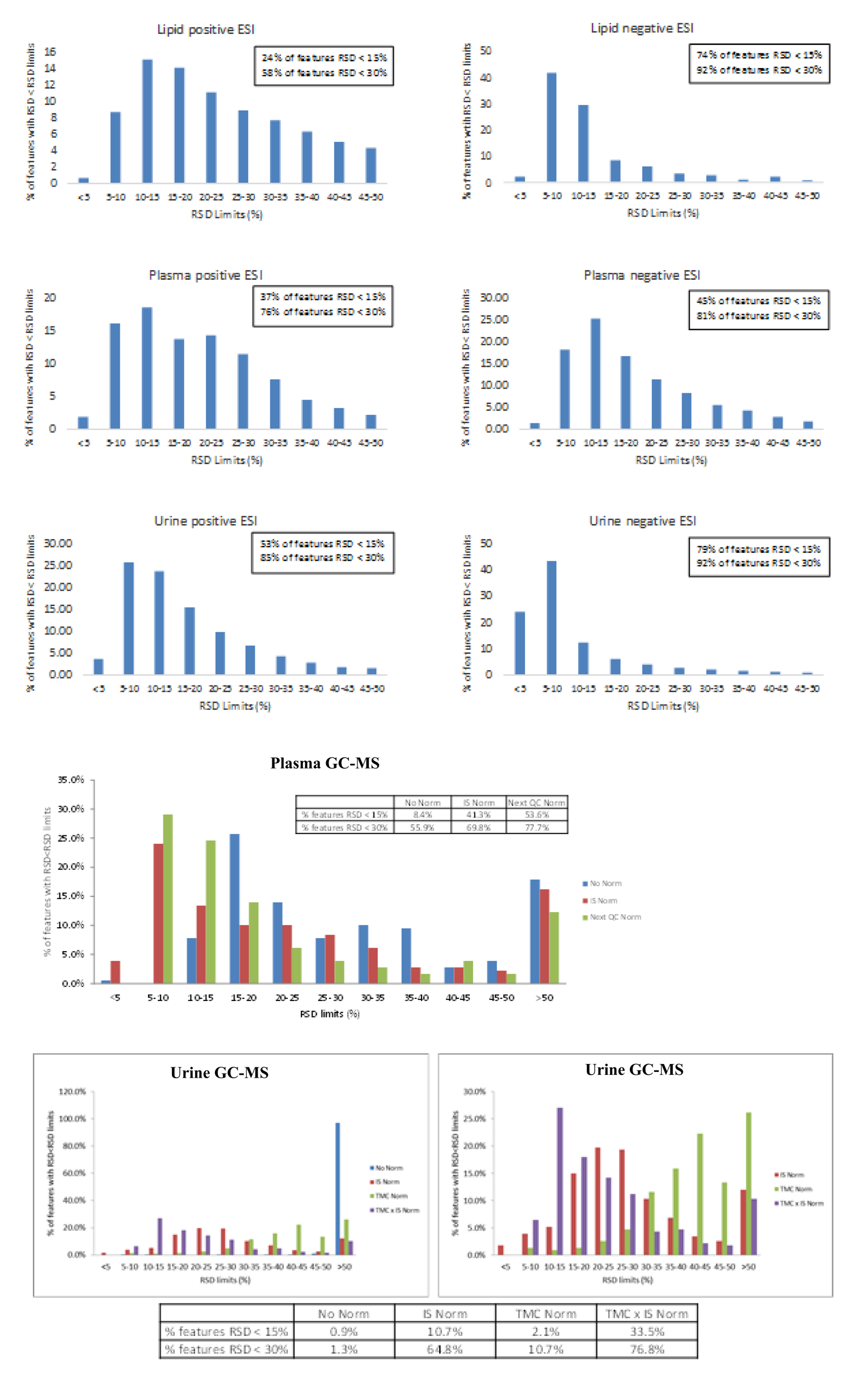

Supplement: S4 Fig — (TIFF) [file pntd.0009999.s004.tiff]
