# Supplementary material for: Multimodal biomarker discovery for active Onchocerca volvulus infection
Source: PLoS Negl Trop Dis. 2021 Nov 29;15(11):e0009999. doi: 10.1371/journal.pntd.0009999 (PMC8659328; doi:10.1371/journal.pntd.0009999)
Supplement: S1 Table — (DOCX) [file pntd.0009999.s005.docx]

**S1 Table.** Overview of samples used in metabolomics and lipidomics discovery study

| **Discipline** | **Matrix** | **Technique** | **NEC (Ov16-)** | **NP (Ov16+)** | **LF** | **QC** | **Blanks** | **Total** |
| --- | --- | --- | --- | --- | --- | --- | --- | --- |
| **Metabolomics** | Urine | RP-LC Q-TOF-MS (+) | 18 | 67 | 8 | 25 | 2 | 120 |
|  | Urine | RP-LC Q-TOF-MS (-) | 18 | 67 | 8 | 25 | 2 | 120 |
|  | Urine | GC-MS | 18 | 67 | 8 | 25 | 2 | 120 |
|  | Plasma | RP-LC Q-TOF-MS (+) | 20 | 68 | 8 | 22 | 2 | 120 |
|  | Plasma | RP-LC Q-TOF-MS (-) | 20 | 68 | 8 | 22 | 2 | 120 |
|  | Plasma | GC-MS | 20 | 68 | 5 | 25 | 2 | 120 |
| **Lipidomics** | Plasma | RP-LC Q-TOF-MS (+) | 20 | 68 | 8 | 22 | 2 | 120 |
|  | Plasma | RP-LC Q-TOF-MS (-) | 20 | 68 | 8 | 22 | 2 | 120 |
