# Supplementary material for: Multimodal biomarker discovery for active Onchocerca volvulus infection
Source: PLoS Negl Trop Dis. 2021 Nov 29;15(11):e0009999. doi: 10.1371/journal.pntd.0009999 (PMC8659328; doi:10.1371/journal.pntd.0009999)
Supplement: S2 Table — (DOCX) [file pntd.0009999.s006.docx]

**S2 Table.** Characteristics of features selected from the comparative plasma lipid profiling study

| ESI | RT  (min) | Mass  (Da) | Formula | Compound name | *p* | *p*_corr_ | FC | RSD  QC (%) | *n%*  NP / CTRL / LF |
| --- | --- | --- | --- | --- | --- | --- | --- | --- | --- |
| + | 0.91 | 362.2052 | C21 H30 O5 | Cortisol (hydrocortisone) | 0.033 | 0.167 | 1.69 | 48.94 | 100 / 100 / 100 |
| + | 15.00 | 681.5908 | C41 H76 O6 | TG(16:0/18:1/4:0) | < 0.001 | 0.004 | 5.05 | 19.20 | 100 / 100 / 100 |
| + | 20.02 | 804.7207 | C51 H96 O6 | TG(16:0/18:1/14:0) | < 0.001 | 0.002 | 2.04 | 21.38 | 100 / 100 / 100 |
| + | 18.74 | 774.6840 | C49 H60 O6 | TG(16:1/18:1/12:0) | 0.007 | 0.064 | 2.04 | 15.28 | 100 / 100 / 100 |
| + | 19.46 | 817.7310 | C51 H94 O6 | TG(16:1/18:1/14:0) | < 0.001 | 0.002 | 2.04 | 16.57 | 100 / 100 / 100 |
| + | 18.01 | 837.6936 | C53 H88 O6 | TG(16:1/12:0/22:6)/ TG(18:1/10:0/22:6) | 0.033 | 0.167 | 1.47 | 13.03 | 100 / 100 / 100 |
| + | 4.28 | 515.2980 | C26 H46 N O7 P | PC(16:1/0:0) | 0.019 | 0.121 | 1.69 | 25.90 | 100 / 100 / 100 |
| + | 9.94 | 783.5778 | C44 H82 N O8 P | PC(36:3) | < 0.001 | 0.007 | 1.08 | 2.80 | 100 / 100 / 100 |
| + | 15.21 | 879.7070 | C52 H98 N O7 P | PCp(44:3) / Pca(44:4) | 0.002 | 0.032 | 1.59 | 13.81 | 100 / 100 / 100 |
| + | 6.05 | 255.2562 | C16 H33 N O | Palmitic amide* | 0.040 | 0.190 | 1.87 | 13.04 | 100 / 100 / 100 |
| + | 5.30 | 236.2140 | C16 H28 O | Hexadecadienal* | 0.023 | 0.136 | 2.85 | 12.29 | 100 / 100 / 100 |
| + | 22.46 | 1156.9609 | C75 H128 O8 | *Unknown* | 0.050 | 0.216 | *Inf.* | - | 24 / 0 / 33 |
| + | 23.59 | 1187.0079 | C77 H134 O8 | *Unknown* | 0.099 | 0.312 | *Inf.* | - | 18 / 0 / 0 |
| + | 16.77 | 728.5960 | C46 H80 O6 | TG(16:0/14:0/ibuprofen) | 0.123 | 0.350 | *Inf.* | 19.22 | 16 / 0 / 0 |
| + | 17.12 | 754.6125 | C48 H82 O6 | TG(16:0/16:1/ibuprofen) | 0.063 | 0.243 | *Inf.* | 19.73 | 22 / 0 / 17 |
| + | 17.33 | 828.6279 | C54 H84 O6 | TG(16:0/22:6/ibuprofen) | 0.152 | 0.391 | *Inf.* | - | 14 / 0 / 0 |
| + | 17.56 | 854.6436 | C56 H86 O6 | TG(18:1/22:6/ibuprofen) | 0.123 | 0.350 | *Inf.* | - | 16 / 0 / 0 |
| + | 17.48 | 780.6290 | C50 H84 O6 | TG(16:1/18:1/ibuprofen) | 0.152 | 0.391 | *Inf.* | 14.16 | 14 / 0 / 0 |
| + | 1.45 | 284.0730 | C16 H13 Cl N2 O | Diazepam | 0.291 | 0.553 | *Inf.* | 10.45 | 8 / 0 / 17 |
| - | 0.56 | 218.0255 | C8 H10 O5 S | Tyrosol sulfate | < 0.001 | 0.003 | 14.37 | 16.65 | 100 / 83 / 100 |
| - | 4.72 | 451.2715 | C21 H42 N O7 P | PE(16:1/0:0) | < 0.001 | 0.004 | 2.20 | 43.92 | 100 / 100 / 100 |
| - | 7.32 | 554.4549 | C33 H62 O6 | *Unknown* | 0.026 | 0.113 | 1.73 | 16.82 | 100 / 100 / 100 |
| - | 18.86 | 810.6743 | C52 H90 O6 | *Unknown* | < 0.001 | < 0.001 | *Inf.* | 64.01 | 100 / 42 / 100 |
| - | 19.02 | 836.6895 | C54 H92 O6 | *Unknown* | 0.001 | 0.008 | 4.14 | 26.19 | 100 / 100 / 100 |
| - | 9.18 | 689.5010 | C37 H72 N O8 P | PE(16:0/16:1) | < 0.001 | < 0.001 | 3.56 | 15.10 | 100 / 100 / 100 |
| - | 8.19 | 782.4952 | C39 H75 O13 P | PI(16:0/14:0) | < 0.001 | < 0.001 | *Inf.* | 13.94 | 100 / 83 / 100 |
| - | 8.35 | 808.5108 | C41 H77 O13 P | PI(16:0/16:1) | < 0.001 | < 0.001 | 3.32 | 9.80 | 100 / 100 / 100 |
| - | 8.17 | 634.4600 | C40 H60 O3 | tetrahydro-§,§-carotene-3,4,4'-triol (formate) | < 0.001 | 0.001 | 5.67 | 13.25 | 100 / 92 / 100 |
| - | 7.35 | 805.5528 | C46 H80 N O8 P | PE(41:6)^1^ | < 0.001 | < 0.001 | 6.65 | 12.53 | 100 / 100 / 100 |
| - | 4.65 | 539.3223 | C25 H50 N O9 P | PC(16:1/0:0) (formate) | < 0.001 | 0.001 | 2.06 | 8.75 | 100 / 100 / 100 |
| - | 7.49 | 695.4742 | C35 H70 N O10 P | PC(12:0/14:0) (formate) | < 0.001 | < 0.001 | *Inf.* | 9.56 | 100 / 0 / 100 |
| - | 9.08 | 776.5450 | C41 H80 N O10 P | PC(16:0/16:1) (formate) | < 0.001 | < 0.001 | 2.89 | 4.95 | 100 / 100 / 100 |
| - | 8.53 | 799.5339 | C43 H78 N O10 P | PC(14:0/20:4) (formate) | < 0.001 | 0.001 | 2.07 | 8.93 | 100 / 100 / 100 |
| - | 8.87 | 751.5358 | C39 H78 N O10 P | PC(16:0/14:0) (formate) | < 0.001 | < 0.001 | 4.78 | 17.40 | 100 / 100 / 100 |
| - | 8.14 | 723.5058 | C37 H74 N O10 P | PC(14:0/14:0)formate / PC(12:0/16:0)formate | < 0.001 | < 0.001 | 6.86 | 10.10 | 100 / 100 / 100 |
| - | 9.52 | 877.5835 | C49 H84 N O10 P | PC(18:1/22:6) (formate) | 0.034 | 0.131 | 1.30 | 5.62 | 100 / 100 / 100 |
| - | 8.30 | 749.5249 | C39 H76 N O10 P | PC(16:1/14:0) (formate) | < 0.001 | < 0.001 | 2.16 | 23.13 | 100 / 100 / 100 |
| - | 11.47 | 832.6625 | C46 H93 N2 O8 P | SM(d18:1/22:0) (formate) | < 0.001 | 0.006 | 2.30 | 7.36 | 100 / 100 / 100 |
| - | 5.91 | 254.2249 | C16 H30 O2 | C16:1* | 0.002 | 0.015 | 3.35 | 10.44 | 100 / 100 / 100 |
| - | 5.18 | 276.2092 | C18 H28 O2 | C18:4* | 0.008 | 0.047 | 1.70 | 11.45 | 100 / 100 / 100 |

*p –* Mann-Whitney unpaired analysis; *p_corr_* – Mann-Whitney unpaired analysis with Benjamini-Hochberg false discovery rate correction; FC – Fold Change; *Inf.* – Infinite upregulation (typically not detected in one sample group); *n%* – percentage of samples in which the lipid is detected; *- no MS/MS fragmentation spectrum is included for this lipid (non-informative MS/MS fragmentation spectrum); ^1^ – identification only based on mass.
