# Supplementary material for: Multimodal biomarker discovery for active Onchocerca volvulus infection
Source: PLoS Negl Trop Dis. 2021 Nov 29;15(11):e0009999. doi: 10.1371/journal.pntd.0009999 (PMC8659328; doi:10.1371/journal.pntd.0009999)
Supplement: S3 Table — (DOCX) [file pntd.0009999.s007.docx]

**S3 Table.** Characteristics of features selected from the comparative LC-MS based plasma metabolite profiling study

| ESI | RT  (min) | Mass  (Da) | Formula | Compound name | *p* | *p*_corr_ | FC | *n%*  NP / CTRL / LF |
| --- | --- | --- | --- | --- | --- | --- | --- | --- |
| + | 3.60 | 381.1730 | C18 H27 N3 O4 S | *unknown* | <0.001 | <0.001 | 8.35 | 100 / 85 / 100 |
| + | 0.99 | 229.0890 | C9 H15 N3 O2 S | Ergothioneine | <0.001 | <0.001 | 5.86 | 100 / 100 / 100 |
| + | 11.48 | 493.3170 | C24 H48 N O7 P | PC(16:1/0:0) | <0.001 | <0.001 | 2.25 | 100 / 100 / 100 |
| + | 11.43 | 451.2710 | C21 H42 N O7 P | PE(16:1/0:0) | <0.001 | <0.001 | 2.96 | 100 / 100 / 100 |
| + | 8.66 | 600.3270 | C34 H48 O9 | C28H40O3 glucuronide | <0.001 | 0.003 | *Inf.* | 65 / 15 / 38 |
| + | 3.24 | 268.0810 | C10 H12 N4 O5 | Inosine | <0.001 | <0.001 | *Inf.* | 90 / 10 / 100 |
| + | 11.52 | 489.3790 | C30 H51 N O4 | *unknown* | <0.001 | <0.001 | *Inf.* | 93 / 70 / 100 |
| + | 4.05 | 151.0630 | C8 H9 N O2 | *p*-acetamidophenol | 0.006 | 0.058 | *Inf.* | 49 / 10 / 25 |
| + | 3.46 | 327.0960 | C14 H17 N O8 | *p*-acetamidophenyl glucuronide | 0.060 | 0.254 | 11.16 | 25 / 5 / 13 |
| + | 9.45 | 341.0220 | C15 H13 Cl2 N O4 | 3-hydroxy-4-methoxydiclofenac | 0.067 | 0.271 | 12.74 | 35 / 15 / 25 |
| + | 8.29 | 202.1720 | C15 H22 | alpha-curcumene | 0.022 | 0.131 | 8.78 | 24 / 0 / 38 |
| + | 2.75 | 222.0720 | C8 H14 O7 | Ethyl glucuronide | 0.032 | 0.169 | *Inf.* | 28 / 5 / 38 |
| + | 9.06 | 658.3210 | C32 H50 O14 | C20H36O3 diglucuronide | 0.003 | 0.037 | *Inf.* | 59 / 25 / 75 |
| + | 7.81 | 447.3340 | C27 H45 N O4 | *unknown* | <0.001 | 0.001 | *Inf.* | 72 / 15 / 38 |
| - | 4.24 | 189.9930 | C6 H6 O5 S | Dihydroxybenzene sulfate | <0.001 | <0.001 | 1.57 | 100 / 100 / 100 |
| - | 10.72 | 386.2430 | C24 H34 O4 | *unknown* | <0.001 | 0.001 | *Inf.* | 72 / 30 / 25 |
| - | 11.24 | 562.2758 | C30 H42 O10 | C24H34O4 glucuronide | <0.001 | 0.001 | *Inf.* | 50 / 0 / 0 |
| - | 9.20 | 738.3090 | C36 H50 O16 | C24H34O4 diglucuronide | 0.007 | 0.027 | *Inf.* | 40 / 10 / 25 |
| - | 14.05 | 548.2980 | C30 H44 O9 | C24H36O3 glucuronide | <0.001 | 0.003 | *Inf.* | 44 / 0 / 13 |
| - | 1.00 | 229.0880 | C9 H15 N3 O2 S | Ergothioneine | <0.001 | <0.001 | *Inf.* | 99 / 40 / 100 |
| - | 2.79 | 222.0740 | C8 H14 O7 | Ethyl glucuronide | 0.003 | 0.014 | *Inf.* | 40 / 5 / 50 |
| - | 3.26 | 268.0810 | C10 H12 N4 O5 | Inosine | <0.001 | <0.001 | *Inf.* | 100 / 100 / 100 |
| - | 3.47 | 327.0950 | C14 H17 N O8 | *p*-acetamidophenyl glucuronide | 0.003 | 0.014 | 7.09 | 99 / 95 / 100 |
| - | 3.83 | 231.0204 | C8 H9 N O5 S | *p*-acetamidophenyl sulfate | <0.001 | <0.001 | *Inf.* | 99 / 75 / 63 |
| - | 11.26 | 539.3220 | C25 H50 N O9 P | PC(0:0/16:1) (formate) | <0.001 | <0.001 | 2.09 | 100 / 100 / 100 |
| - | 11.49 | 539.3220 | C25 H50 N O9 P | PC(16:1/0:0) (formate) | <0.001 | <0.001 | 2.01 | 100 / 100 / 100 |
| - | 6.52 | 470.0870 | C20 H22 O11 S | C14H14O2 glucuronide sulfate | <0.001 | <0.001 | *Inf.* | 78 / 10 / 75 |
| - | 3.33 | 425.1510 | C16 H27 N O12 | Dimethyl 2-acetamido-4-O-α-L-allopyranosyl-2-deoxy-α-L-gulopyranosiduronate | 0.001 | 0.004 | *Inf.* | 41 / 0 / 50 |
| - | 1.17 | 125.9990 | C2 H6 O4 S | Ethyl hydrogen sulfate | <0.001 | <0.001 | 5.97 | 100 / 100 / 100 |
| - | 3.15 | 261.0310 | C9 H11 N O6 S | Tyrosine O-sulfate | 0.004 | 0.017 | 1.28 | 100 / 100 / 100 |
| - | 8.14 | 414.1547 | C19 H26 O10 | C13H18O4 glucuronide | <0.001 | <0.001 | *Inf.* | 54 / 0 / 25 |
| - | 11.26 | 366.2073 | C17 H34 O6 S | C17H34O3 sulfate | <0.001 | <0.001 | *Inf.* | 99 / 10 / 63 |
| - | 6.52 | 470.0866 | C20 H22 O11 S | C14H14O2 glucuronide sulfate | 0.001 | 0.007 | *Inf.* | 59 / 20 / 88 |
| - | 8.17 | 246.0568 | C10 H14 O5 S | C10H14O2 sulfate | <0.001 | <0.001 | *Inf.* | 62 / 0 / 63 |
| - | 2.02 | 136.0385 | C5 H4 N4 O | hypoxanthine | <0.001 | <0.001 | 3.27 | 100 / 100 / 100 |
| - | 15.07 | 414.3337 | C24 H46 O5 | *unknown* | 0.002 | 0.009 | 2.88 | 100 / 95 / 100 |
| - | 8.15 | 626.3177 | C28 H50 O15 | C22H42O9 glucuronide | <0.001 | 0.002 | *Inf.* | 56 / 10 / 88 |
| - | 9.06 | 602.3373 | C29 H50 N2 O11 | C23H42N2O5 glucuronide | 0.008 | 0.030 | 2.18 | 96 / 100 / 100 |
| - | 7.69 | 226.1200 | C12 H18 O4 | 3,4-methylenesebacic acid (tentative ID) | 0.016 | 0.052 | 5.48 | 72 / 60 / 38 |
| - | 9.00 | 230.1518 | C12 H22 O4 | dodecanedioic acid (tentative ID) | 0.012 | 0.041 | 1.95 | 100 / 100 / 100 |
| - | 3.35 | 496.6813 |  | *Unknown peptide** | 0.002 | 0.008 | *Inf.* | 54 / 20 / 50 |
| - | 15.20 | 236.2140 | C16 H28 O | Hexadecadienal* | 0.001 | 0.006 | 3.03 | 100 / 100 / 100 |
| - | 13.57 | 300.2663 | C18 H36 O3 | C18:0-OH* | <0.001 | <0.001 | 3.10 | 100 / 100 / 100 |
| - | 3.53 | 455.1895 | C20 H29 N3 O9 | *unknown* | 0.016 | 0.052 | 13.07 | 60 / 35 / 75 |
| - | 1.00 | 126.0257 | C5 H6 N2 S | *unknown* | <0.001 | <0.001 | 5.94 | 100 / 100 / 100 |
| - | 8.38 | 591.3229 | C28 H49 N O12 | C22H41NO6 glucuronide | 0.002 | 0.010 | 4.00 | 85 / 80 / 100 |
| - | 8.86 | 713.3759 | C32 H60 N O14 P | *Unknown lipid** | <0.001 | 0.003 | *Inf.* | 44 / 0 / 13 |
| - | 9.21 | 715.3867 | C32 H62 N O14 P | *Unknown lipid** | 0.001 | 0.006 | *Inf.* | 62 / 30 / 50 |
| - | 10.83 | 790.4194 | C35 H66 O19 | *unknown** | 0.004 | 0.017 | 7.72 | 99 / 85 / 100 |

*p –* Mann-Whitney unpaired analysis; *p_corr_* – Mann-Whitney unpaired analysis with Benjamini-Hochberg false discovery rate correction; FC – Fold Change; *Inf.* – Infinite upregulation (not detected in one sample group); *n%* – percentage of samples in which the metabolite is detected; *- no MS/MS fragmentation spectrum is included for this metabolite (non-informative MS/MS fragmentation spectrum).
