# Supplementary material for: Multimodal biomarker discovery for active Onchocerca volvulus infection
Source: PLoS Negl Trop Dis. 2021 Nov 29;15(11):e0009999. doi: 10.1371/journal.pntd.0009999 (PMC8659328; doi:10.1371/journal.pntd.0009999)
Supplement: S4 Table — (DOCX) [file pntd.0009999.s008.docx]

**Table S4.** Characteristics of features selected from the comparative LC-MS based urine metabolite profiling study

| ESI | RT  (min) | Mass  (Da) | Formula | Compound name | *p* | *p*_corr_ | FC | RSD  QC (%) | *n%*  NP / CTRL / LF |
| --- | --- | --- | --- | --- | --- | --- | --- | --- | --- |
| + | 3.42 | 895.3533 | C35 H61 N O25 | *unknown* | 0.012 | 0.084 | *Inf.* | 12.27% | 28 / 0 / 0 |
| + | 3.59 | 270.0674 | C11 H14 N2 O4 S | 3-cysteinylacetaminophen | 0.013 | 0.089 | 3.13 | 3.60% | 99 / 100 / 100 |
| + | 5.95 | 205.0739 | C11 H11 N O3 | Cinnamoylglycine | <0.001 | <0.001 | *Inf.* | 6.84% | 99 / 0 / 0 |
| + | 1.25 | 125.9987 | C2 H6 O4 S | Ethyl hydrogen sulfate | 0.004 | 0.045 | *Inf.* | 24.21% | 34 / 0 / 50 |
| + | 3.97 | 247.1056 | C10 H17 N O6 | *unknown* | 0.006 | 0.053 | *Inf.* | - | 33 / 0 / 0 |
| - | 5.97 | 205.0740 | C11 H11 N O3 | Cinnamoylglycine | <0.001 | <0.001 | *Inf.* | 4.53 | 93 / 72 / 38 |
| - | 4.05 | 157.9440 | C2 H3 Cl O4 S | 2-Chloro-2-oxoethane-1-sulfonic acid | 0.029 | 0.111 | *Inf.* | 3.98 | 22 / 0 / 0 |
| - | 11.14 | 366.2070 | C17 H34 O6 S | C17H34O3 sulfate | <0.001 | 0.005 | *Inf.* | 11.62 | 49 / 0 / 13 |
| - | 4.32 | 275.0463 | C10 H13 N O6 S | Tyrosine methyl ester 4-sulfate | 0.010 | 0.053 | 1.81 | 2.31 | 100 / 100 / 100 |
| - | 6.24 | 218.0249 | C8 H10 O5 S | Tyrosol 4-sulfate | <0.001 | <0.001 | 9.85 | 5.88 | 100 / 100 / 100 |
| - | 7.24 | 390.1309 | C20 H22 O8 | C14H14O2 glucuronide | <0.001 | <0.001 | *Inf.* | 4.95 | 88 / 56 / 88 |
| - | 6.49 | 470.0880 | C20 H22 O11 S | C14H14O2 glucuronide sulfate | <0.001 | 0.006 | 31.52 | 6.78 | 99 / 83 / 100 |
| - | 7.67 | 249.9959 | C8 H10 O5 S2 | C8H10O2S sulfate | <0.001 | <0.001 | *Inf.* | 6.33 | 90 / 61 / 100 |
| - | 8.50 | 298.1205 | C18 H18 O4 | Vitamin K1 aglycone I | 0.001 | 0.013 | 7.11 | 12.46 | 100 / 100 / 100 |
| - | 8.97 | 382.1618 | C19 H26 O8 | 1-(alpha-Methyl-4-(2-methylpropyl)benzeneacetate)-beta-D-Glucopyranuronic acid | 0.047 | 0.153 | 10.37 | 13.67 | 27 / 6 / 25 |
| - | 5.06 | 153.0194 | C3 H8 N O4 P | vanillin 4-sulfate | <0.001 | 0.001 | *Inf.* | 7.34 | 85 / 17 / 50 |
| - | 5.94 | 247.9991 | C8 H8 O7 S | vanillic acid 4-sulfate | 0.001 | 0.012 | 3.79 | 5.61 | 100 / 100 / 100 |
| - | 1.60 | 148.0362 | C5 H8 O5 | hydroxyglutaric acid | 0.005 | 0.032 | 1.13 | 14.82 | 97 / 100 / 100 |
| - | 3.94 | 310.0195 | C10 H14 O7 S2 | *unknown* | <0.001 | <0.001 | *Inf.* | 5.21 | 99 / 44 / 63 |
| - | 6.24 | 322.0182 | C11 H14 O7 S2 | *unknown* | 0.001 | 0.011 | 4.45 | 9.38 | 91 / 89 / 88 |
| - | 2.89 | 344.1123 | C15 H20 O9 | C9H12O3 glucuronide | <0.001 | 0.005 | *Inf.* | 6.29 | 54 / 6 / 38 |
| - | 8.90 | 276.0906 | C17 H12 N2 O2 | *unknown** | 0.015 | 0.071 | 13.38 | 9.98 | 27 / 0 / 0 |
| - | 4.70 | 277.0074 | C9 H11 N O7 S | C9H11NO4 sulfate | 0.004 | 0.030 | 25.79 | 7.65 | 97 / 83 / 88 |
| - | 2.42 | 165.0760 | C9 H11 N O2 | *unknown** (no phenylalanine) | <0.001 | 0.001 | *Inf.* | 11.32 | 63 / 6 / 25 |
| - | 3.33 | 1213.4580 |  | *unknown peptide** | 0.012 | 0.063 | 19.67 | 12.65 | 51 / 22 / 75 |
| - | 1.27 | 125.9990 | C2 H6 O4 S | Ethyl hydrogen sulfate | <0.001 | <0.001 | 13.19 | 2.92 | 100 / 100 / 100 |
| - | 2.74 | 222.0740 | C8 H14 O7 | Ethyl glucuronide | <0.001 | 0.002 | *Inf.* | 3.21 | 90 / 50 / 88 |
| - | 1.70 | 288.0513 | C8 H16 O9 S | Ethyl glucopyranoside sulfate | 0.004 | 0.032 | *Inf.* | 6.23 | 34 / 0 / 50 |
| - | 4.06 | 261.0301 | C9 H11 N O6 S | 2-methoxyacetaminophen sulfate | 0.005 | 0.034 | 6.84 | 1.70 | 100 / 94 / 100 |
| - | 9.19 | 382.1620 | C19 H26 O8 | Ibuprofen glucuronide | <0.001 | 0.002 | *Inf.* | 6.94 | 88 / 28 / 100 |

*p –* Mann-Whitney unpaired analysis; *p_corr_* – Mann-Whitney unpaired analysis with Benjamini-Hochberg FDR correction; FC – Fold Change*; Inf.* – Infinite upregulation (typically not detected in one sample group); *n%* – percentage of samples in which the metabolite is detected; *- no MS/MS fragmentation spectrum is included for this metabolite (non-informative MS/MS fragmentation spectrum).
