# Supplementary material for: Multimodal biomarker discovery for active Onchocerca volvulus infection
Source: PLoS Negl Trop Dis. 2021 Nov 29;15(11):e0009999. doi: 10.1371/journal.pntd.0009999 (PMC8659328; doi:10.1371/journal.pntd.0009999)
Supplement: S5 Table — (DOCX) [file pntd.0009999.s009.docx]

**S5 Table.** Characteristics of features selected from the comparative GC-MS based plasma metabolite profiling study

| RT (min) | Mass (Da) | [Nodule positive] Vs [Non-endemic control] | | | | [Nodule positive] Vs [LF infected] | | | | [LF infected] Vs [Non-endemic control] | | | | Identification | Score | Lib | RSD  QC (%) |
| --- | --- | --- | --- | --- | --- | --- | --- | --- | --- | --- | --- | --- | --- | --- | --- | --- | --- |
|  |  | *p* | *p*_corr_ | FC (abs) | Reg | *p* | *p*_corr_ | FC (abs) | Reg | *p* | *p*_corr_ | FC (abs) | Reg |  |  |  |  |
| 24.67 | 361 | <0.001 | 0.001 | 3.44 | down |  |  |  |  |  |  |  |  | [6255] maltose  2 [24.915] | 76.23 | Fiehn | 8.5 |
| 16.47 | 265 | <0.001 | <0.001 | 3.28 | up |  |  |  |  | 0.001 | 0.007 | 8.50 | up | [790] hypoxanthine [16.479] | 79.2 | Fiehn+NIST | 13.7 |
| 18.72 | 311 | 0.002 | 0.018 | 2.33 | up |  |  |  |  |  |  |  |  | [445638] palmitoleic acid [18.728] | 91.06 | Fiehn | 10.1 |
| 14.79 | 257 | <0.001 | 0.002 | 2.32 | up |  |  |  |  |  |  |  |  | [3893] lauric acid [14.789] | 87.95 | Fiehn | 5.7 |
| 11.05 | 292 | <0.001 | 0.003 | 1.86 | down |  |  |  |  |  |  |  |  | (R*,S*)-2,3-Dihydroxybutanoic acid | 65.11 | NIST | 6.4 |
| 30.25 | 311 | <0.001 | <0.001 | 1.85 | up |  |  |  |  |  |  |  |  | 2,2-Dimethylpentan-3-ol, dimethylpentafluorophenylsilyl ether | 67.98 | NIST | 15.1 |
| 16.21 | 299 | <0.001 | <0.001 | 1.74 | down | 0.002 | 0.009 | 1.49 | up | 0.004 | 0.025 | 2.59 | down | [1015] O-phosphocolamine [16.232] | 88.12 | Fiehn+NIST | 9.6 |
| 16.12 | 217 | <0.001 | 0.003 | 1.61 | up |  |  |  |  |  |  |  |  | 1,5-Anhydro-D-sorbitol | 77.73 | NIST | 13.5 |
| 20.39 | 202.1 | <0.001 | 0.002 | 1.61 | down | 0.009 | 0.043 | 1.75 | down |  |  |  |  | [6305] L-tryptophan 2 [20.466] | 74.94 | Fiehn | 12.9 |
| 12.06 | 248 | <0.001 | 0.001 | 1.53 | down |  |  |  |  |  |  |  |  | [239] Beta- alanine 1 [12.044] | 62.01 | Fiehn+NIST | 8.6 |
| 31.24 | 297 | <0.001 | 0.005 | 1.51 | down |  |  |  |  |  |  |  |  | 5-Methoxysalicylic acid | 82.49 | NIST | 15.4 |
| 7.76 | 72 | <0.001 | 0.001 | 1.46 | down | 0.001 | 0.009 | 1.49 | down |  |  |  |  | [6287] L-valine 1 [7.296] | 97.52 | Fiehn | 7.1 |
| 28.32 | 382 | 0.005 | 0.040 | 1.43 | down |  |  |  |  |  |  |  |  | Campesterol | 68.64 | NIST | 11.1 |
| 9.30 | 144 | <0.001 | 0.004 | 1.43 | down | 0.008 | 0.036 | 1.50 | down |  |  |  |  | [6287] L-valine 2 [9.151] | 96.98 | Fiehn+NIST | 19.4 |
| 10.76 | 292 | 0.006 | 0.041 | 1.41 | up |  |  |  |  |  |  |  |  | [439194] glyceric acid [10.735] | 95.09 | Fiehn | 6.2 |
| 15.96 | 357 | 0.002 | 0.018 | 1.35 | down |  |  |  |  |  |  |  |  | [754] glycerol 1-phosphate [16.056] | 97.42 | Fiehn+NIST | 21.0 |
| 32.48 | 309 | 0.003 | 0.029 | 1.33 | down |  |  |  |  |  |  |  |  | 2,6-Diphenyl-1,7-dihydrodipyrrolo[2,3-b:3',2'-e]pyridine | 72.86 | NIST | 18.2 |
| 13.58 | 120 | <0.001 | <0.001 | 1.33 | down |  |  |  |  |  |  |  |  | [994] Phenylalanine 1 [13.545] | 94.51 | Fiehn | 5.7 |
| 9.72 | 189 | 0.004 | 0.029 | 1.33 | down | 0.011 | 0.047 | 1.67 | down |  |  |  |  | [1176] urea [9.599] | 98.81 | Fiehn | 13.8 |
| 14.48 | 218 | <0.001 | 0.002 | 1.30 | down | 0.007 | 0.033 | 1.46 | down |  |  |  |  | L-phenylalanine | 97.72 | NIST | 13.8 |
| 21.46 | 217 | <0.001 | 0.002 | 1.28 | up |  |  |  |  |  |  |  |  | Pseudo uridine | 78.94 | NIST | 9.0 |
| 10.50 | 174 | 0.003 | 0.025 | 1.27 | up |  |  |  |  |  |  |  |  | [750] glycine [10.456] | 98.75 | Fiehn | 6.3 |

*p –* Mann-Whitney unpaired analysis; *p_corr_* – Mann-Whitney unpaired analysis with Benjamini-Hochberg false discovery rate correction; *FC* – Fold Change; *Reg* – Regulation; *Score* – Match factor against Mass spectral library; *Fiehn* – Fiehn library; *NIST* – NIST11 library.
