# Supplementary material for: Multimodal biomarker discovery for active Onchocerca volvulus infection
Source: PLoS Negl Trop Dis. 2021 Nov 29;15(11):e0009999. doi: 10.1371/journal.pntd.0009999 (PMC8659328; doi:10.1371/journal.pntd.0009999)
Supplement: S6 Table — (DOCX) [file pntd.0009999.s010.docx]

**S6 Table**. Characteristics of features selected from the comparative GC-MS based urine metabolite profiling study

| RT (min) | Mass (Da) | [Nodule positive] Vs [Non-endemic control] | | | | [Nodule positive] Vs [LF infected] | | | | [LF infected] Vs [Non-endemic control] | | | | Identification | Score | Lib | RSD QC (%) |
| --- | --- | --- | --- | --- | --- | --- | --- | --- | --- | --- | --- | --- | --- | --- | --- | --- | --- |
|  |  | *p* | *p*_corr_ | FC (abs) | Reg | *p* | *p*_corr_ | FC (abs) | Reg | p | *p*_corr_ | FC (abs) | Reg |  |  |  |  |
| 26.81 | 375.2 | 0.003 | 0.033 | 4.70 | down |  |  |  |  |  |  |  |  | Unknown |  |  | 14.4 |
| 23.89 | 361 | <0.001 | 0.003 | 4.48 | down |  |  |  |  |  |  |  |  | [5988] Sucrose [23.988] | 86.41 | Fiehn | 16.8 |
| 16.95 | 267 | <0.001 | 0.002 | 4.00 | up | <0.001 | 0.012 | 15.607759 | up |  |  |  |  | [464] hippuric acid 2 [16.87] | 83.83 | Fiehn+NIST | 19.0 |
| 17.14 | 307.2 | 0.001 | 0.013 | 3.88 | down |  |  |  |  |  |  |  |  | [5984] fructose 2 [17.288] | 73.17 | Fiehn+NIST | 12.4 |
| 18.41 | 333 |  | 0.001 | 3.18 | down |  |  |  |  |  |  |  |  | [604] gluconic acid 2 [18.297] | 78.98 | Fiehn+NIST | 29.9 |
| 25.61 | 361.2 | 0.004 | 0.036 | 3.07 | up |  |  |  |  | <0.001 | 0.018 | Infinite | up | [439193] isomaltose 1 [25.625] | 82.83 | Fiehn | 12.6 |
| 19.85 | 294 | 0.001 | 0.013 | 2.93 | up | <0.001 | 0.018 | 10.330712 | up |  |  |  |  | 2-hydroxyhippurate | 78.79 | NIST | 10.1 |
| 8.40 | 165 | 0.001 | 0.013 | 2.78 | up |  |  |  |  |  |  |  |  | [2879] p-cresol [8.211] | 82.29 | Fiehn+NIST | 24.2 |
| 17.09 | 307.2 |  | 0.002 | 2.73 | down |  |  |  |  |  |  |  |  | [5984] fructose 1 [17.18] | 77.3 | Fiehn+NIST | 12.5 |
| 10.65 | 254 | <0.001 | 0.001 | 2.61 | up |  |  |  |  |  |  |  |  | [289] catechol [10.524] | 78.04 | Fiehn | 26.2 |
| 11.48 | 218 | 0.004 | 0.036 | 2.39 | down |  |  |  |  |  |  |  |  | [6288] L-threonine 2 [11.464] | 79.26 | Fiehn+NIST | 51.1 |
| 11.05 | 292.1 | <0.001 | 0.008 | 2.27 | down |  |  |  |  |  |  |  |  | (R*,S*)-2,3-Dihydroxybutanoic acid | 86.73 | NIST | 21.0 |
| 9.90 | 174 | 0.001 | 0.011 | 2.12 | down |  |  |  |  |  |  |  |  | Ethanolamine | 94.92 | NIST | 59.8 |
| 10.60 | 147 | <0.001 | 0.003 | 2.07 | up |  |  |  |  |  |  |  |  | [1110] succinic acid [10.509] | 93.2 | Fiehn | 23.5 |
| 14.79 | 335 | 0.001 | 0.013 | 1.84 | down |  |  |  |  |  |  |  |  | 2-Deoxyribonic acid | 84.01 | NIST | 16.3 |
| 16.17 | 292 | 0.002 | 0.021 | 1.66 | down |  |  |  |  |  |  |  |  | 2-Keto-l-gluconic acid | 83.98 | NIST | 15.2 |

*p –* Mann-Whitney unpaired analysis; *p_corr_* – Mann-Whitney unpaired analysis with Benjamini-Hochberg false discovery rate correction; *FC* – Fold Change; *Reg* – Regulation; *Score* – Match factor against Mass spectral library; *Fiehn* – Fiehn library; *NIST* – NIST11 library.
