# Supplementary material for: Multimodal biomarker discovery for active Onchocerca volvulus infection
Source: PLoS Negl Trop Dis. 2021 Nov 29;15(11):e0009999. doi: 10.1371/journal.pntd.0009999 (PMC8659328; doi:10.1371/journal.pntd.0009999)
Supplement: S7 Table — (DOCX) [file pntd.0009999.s011.docx]

**S7 Table.** Overview of study population

| **Characteristic** | **Group** | | |  | | |  | |  |  |  |  |
| --- | --- | --- | --- | --- | --- | --- | --- | --- | --- | --- | --- | --- |
|  | **Nodule positive Ghana** | | **Endemic controls Ghana** | **Non-endemic controls Ghana** | **Lymphatic filariasis Ghana** | | **Healthy Controls Belgium** | **Non-endemic controls Kenya** |  |  |  |  |
| No. of subjects | 98 | | 51 | 54 | 50 | | 50 | 476 |  |  |  |  |
| Age, median (Min-Max) | 47 (21–85) | | 35 (18–81) | 23 (18–56) | 33 (18–68) | | 40.5 (23-59) | 11 (9-14) |  |  |  |  |
| Gender, n (%) |  | |  |  |  | |  |  |  |  |  |  |
| Male | 53 (53) | | 26 (51) | 36 (67) | 36 (72) | | 22 (44) | 238 (50) |  |  |  |  |
| Female | 45 (47) | | 25 (49) | 18 (33) | 14 (28) | | 28 (56) | 238 (50) |  |  |  |  |
| No. of nodules, median (Min-Max) | 1 (1–5) | | 0 | na | na | | na | na |  |  |  |  |
| mf status, n (%) |  | |  |  |  | |  |  |  |  |  |  |
| 0 mf/mg | 87 (89) | | 51 (100) | na | na | | na | na |  |  |  |  |
| 0–5 mf/mg | 10 (9) | | 0 (0) | na | na | | na | na |  |  |  |  |
| 5–10 mf/mg | 1 (1) | | 0 (0) | na | na | | na | na |  |  |  |  |
| No. of IVM rounds, median (Min-Max) | 2 (0–10) | | 0 (0-1) | na | na | | na | na |  |  |  |  |
| Time since last ivermectin treatment, n (%) | |  | | | |  | | |  |  |  | na |
| Not treated | 17 (17) | | 34 (67) | na | na | | na | na |  |  |  |  |
| < 20 months | 67 (68) | | 5 (10) | na | na | | na | na |  |  |  |  |
| > 20 months | 14 (14) | | 12 (24) | na | na | | na | na |  |  |  |  |
| Ov16 status, n (%) |  | |  |  |  | |  |  |  |  |  |  |
| Positive | 68 (69) | | 26 (51) | 9 (17) | 12 (24) | | 0 (0) | 4 (1) |  |  |  |  |
| Negative | 30 (31) | | 25 (49) | 45 (83) | 38 (75) | | 50 (100) | 470 (99) |  |  |  |  |
