# Supplementary material for: Multimodal biomarker discovery for active Onchocerca volvulus infection
Source: PLoS Negl Trop Dis. 2021 Nov 29;15(11):e0009999. doi: 10.1371/journal.pntd.0009999 (PMC8659328; doi:10.1371/journal.pntd.0009999)
Supplement: S8 Table — (DOCX) [file pntd.0009999.s012.docx]

**S8 Table.** Targeted validity verification of LC-MS based metabolomics and lipidomics

| Approach | Matrix | Compound name | RT | *Area*  *(AUC)* | | | Mass accuracy  (ppm) | |
| --- | --- | --- | --- | --- | --- | --- | --- | --- |
|  |  |  | RSD (%) | Avg | Stdev | RSD (%) | Avg | *Stdev* |
| Lipidomics ESI+ | Plasma | PC(16:0/16:0) | 0.065 | 7.23E+07 | 2.90E+06 | 4.01 | 4.67 | 2.77 |
| Lipidomics ESI+ | Plasma | PC(16:0/20:4) | 0.090 | 8.95E+07 | 4.06E+06 | 4.53 | 3.87 | 2.32 |
| Lipidomics ESI+ | Plasma | PCp(34:2) | 0.084 | 7.05E+07 | 4.95E+06 | 7.02 | 4.71 | 2.81 |
| Lipidomics ESI+ | Plasma | PE(16:0/18:1) | 0.064 | 1.65E+06 | 3.28E+05 | 19.91 | 4.82 | 2.75 |
| Lipidomics ESI+ | Plasma | PE(18:0/20:4) | 0.065 | 1.74E+07 | 3.14E+06 | 18.04 | 4.85 | 2.65 |
| Lipidomics ESI+ | Plasma | PE(p16:0/18:1) | 0.090 | 1.90E+06 | 3.76E+05 | 19.75 | 4.75 | 2.81 |
| Lipidomics ESI+ | Plasma | PE(p16:0/20:4) | 0.111 | 5.02E+06 | 5.41E+05 | 10.77 | 4.62 | 2.49 |
| Lipidomics ESI+ | Plasma | PI(16:0/18:1) (NH3) | 0.090 | 5.66E+05 | 1.33E+05 | 23.46 | 4.78 | 2.90 |
| Lipidomics ESI+ | Plasma | PI(18:0/20:4) (NH3) | 0.263 | 2.72E+05 | 1.12E+05 | 41.22 | 4.11 | 3.01 |
| Lipidomics ESI+ | Plasma | PC(22:5/0:0) | 0.052 | 6.67E+06 | 2.12E+06 | 31.82 | 4.50 | 2.58 |
| Lipidomics ESI+ | Plasma | PE(16:0/0:0) | 0.074 | 5.92E+05 | 2.34E+05 | 39.51 | 4.86 | 3.01 |
| Lipidomics ESI+ | Plasma | DG(32:0) (NH3) | 0.075 | 2.48E+06 | 5.76E+05 | 23.24 | 4.94 | 3.20 |
| Lipidomics ESI+ | Plasma | DG(38:5) (NH3) | 0.080 | 3.51E+06 | 5.47E+05 | 15.56 | 4.85 | 2.79 |
| Lipidomics ESI+ | Plasma | cholesterol (-H2O) | 0.132 | 2.57E+07 | 4.83E+06 | 18.80 | 5.16 | 3.46 |
| Lipidomics ESI+ | Plasma | TG(52:1) (NH3) | 0.040 | 1.44E+08 | 8.86E+06 | 6.17 | 2.63 | 2.99 |
| Lipidomics ESI+ | Plasma | SM(d18:1/18:0) | 0.089 | 7.78E+07 | 3.84E+06 | 4.93 | 4.74 | 2.80 |
| Lipidomics ESI+ | Plasma | Cer(d18:1/16:0) | 0.092 | 1.73E+05 | 3.20E+04 | 18.47 | 4.79 | 2.76 |
| Lipidomics ESI+ | Plasma | HexCer(d18:1/18:0) | 0.088 | 6.71E+07 | 4.96E+06 | 7.40 | 4.82 | 2.72 |
| Lipidomics ESI+ | Plasma | HexHexCer(d18:1/24:0) | 0.090 | 3.46E+05 | 5.90E+04 | 17.02 | 4.89 | 2.90 |
| Lipidomics ESI+ | Plasma | HexNAcHexHexHexCer(d18:1/18:0) | 0.106 | 3.34E+04 | 7.11E+03 | 21.25 | 4.54 | 2.60 |
| Lipidomics ESI+ | Plasma | NeuAcHexHexCer(d18:1/18:0) | 0.215 | 1.35E+05 | 2.59E+04 | 19.26 | 4.40 | 2.81 |
| Lipidomics ESI- | Plasma | PC(16:0/16:0) (formate) | 0.076 | 3.75E+06 | 7.99E+05 | 21.31 | 1.20 | 0.71 |
| Lipidomics ESI- | Plasma | PC(16:0/20:4) (formate) | 0.077 | 4.24E+07 | 8.50E+06 | 20.07 | 0.84 | 0.76 |
| Lipidomics ESI- | Plasma | PCp(34:2) (formate) | 0.076 | 2.24E+06 | 6.87E+05 | 30.64 | 0.67 | 0.78 |
| Lipidomics ESI- | Plasma | PE(16:0/18:1) | 0.232 | 1.69E+06 | 5.34E+05 | 31.63 | 0.77 | 0.86 |
| Lipidomics ESI- | Plasma | PE(18:0/20:4) | 0.184 | 1.23E+07 | 2.21E+06 | 18.01 | 0.93 | 0.87 |
| Lipidomics ESI- | Plasma | PE(p16:0/18:1) | 0.099 | 1.34E+06 | 5.07E+05 | 37.82 | 0.62 | 0.72 |
| Lipidomics ESI- | Plasma | PE(p16:0/20:4) | 0.081 | 2.66E+07 | 9.20E+06 | 34.63 | 0.71 | 0.72 |
| Lipidomics ESI- | Plasma | PI(16:0/18:1) | 0.750 | 2.79E+06 | 1.05E+06 | 37.60 | 0.79 | 1.04 |
| Lipidomics ESI- | Plasma | PI(18:0/20:4) | 0.697 | 5.43E+07 | 1.21E+07 | 22.26 | 0.77 | 0.82 |
| Lipidomics ESI- | Plasma | PS(38:4) | 0.088 | 8.79E+05 | 1.79E+05 | 20.38 | 0.59 | 0.57 |
| Lipidomics ESI- | Plasma | PC(16:0/0:0) (formate) | 0.085 | 5.38E+07 | 1.52E+07 | 28.27 | 0.98 | 1.13 |
| Lipidomics ESI- | Plasma | PC(22:5/0:0) (formate) | 0.114 | 1.12E+05 | 4.26E+04 | 38.04 | 1.02 | 0.83 |
| Lipidomics ESI- | Plasma | PE(16:0/0:0) | 0.063 | 1.64E+06 | 9.85E+05 | 60.22 | 0.76 | 0.69 |
| Lipidomics ESI- | Plasma | PE(22:5/0:0) | 0.102 | 1.04E+05 | 4.27E+04 | 40.90 | 0.89 | 0.64 |
| Lipidomics ESI- | Plasma | SM(d18:1/18:0) (formate) | 0.086 | 8.04E+06 | 2.12E+06 | 26.37 | 0.97 | 0.89 |
| Lipidomics ESI- | Plasma | Cer(d18:1/16:0) (formate) | 0.074 | 1.01E+07 | 1.32E+06 | 13.10 | 0.59 | 0.64 |
| Lipidomics ESI- | Plasma | HexCer(d18:1/18:0) (formate) | 0.084 | 1.43E+06 | 3.78E+05 | 26.44 | 0.66 | 0.87 |
| Lipidomics ESI- | Plasma | HexHexCer(d18:1/24:0) (formate) | 0.098 | 2.61E+05 | 4.09E+04 | 15.71 | 1.15 | 1.08 |
| Lipidomics ESI- | Plasma | HexNAcHexHexHexCer(d18:1/18:0) | 0.124 | 8.41E+03 | 1.43E+03 | 17.04 | 1.68 | 1.46 |
| Lipidomics ESI- | Plasma | NeuAcHexHexCer(d18:1/18:0) | 0.484 | 1.38E+05 | 3.57E+04 | 25.93 | 1.20 | 1.26 |
| Lipidomics ESI- | Plasma | C18:0 | 0.292 | 2.39E+07 | 2.71E+06 | 11.32 | 0.55 | 0.50 |
| Lipidomics ESI- | Plasma | C20:5 | 0.120 | 1.46E+06 | 5.14E+05 | 35.13 | 0.65 | 0.73 |
| Metabolomics ESI+ | Plasma | Creatinine | 0.240 | 1.93E+07 | 8.76E+05 | 4.54 | 1.46 | 0.85 |
| Metabolomics ESI+ | Plasma | Tryptophan | 0.098 | 1.20E+07 | 1.02E+06 | 8.49 | 2.20 | 2.55 |
| Metabolomics ESI+ | Plasma | Hippuric acid | 0.069 | 2.01E+05 | 2.38E+04 | 11.84 | 2.88 | 3.57 |
| Metabolomics ESI+ | Plasma | Caffeine | 0.047 | 3.97E+06 | 5.63E+05 | 14.20 | 1.91 | 4.21 |
| Metabolomics ESI+ | Plasma | Phenylacetamide | 0.081 | 5.02E+04 | 5.56E+03 | 11.06 | 2.52 | 2.06 |
| Metabolomics ESI+ | Plasma | Pantothenic Acid | 0.082 | 1.90E+05 | 2.77E+04 | 14.59 | 5.18 | 5.26 |
| Metabolomics ESI+ | Plasma | Uric acid | 0.701 | 5.70E+07 | 4.60E+06 | 8.07 | 4.08 | 4.58 |
| Metabolomics ESI+ | Plasma | Acetylcarnitine | 0.737 | 5.15E+07 | 7.40E+06 | 14.36 | 2.95 | 5.17 |
| Metabolomics ESI+ | Plasma | Methyladenosine | 0.903 | 8.04E+05 | 1.10E+05 | 13.68 | 3.75 | 3.35 |
| Metabolomics ESI+ | Plasma | Phenylalanine | 0.082 | 2.55E+07 | 1.43E+06 | 5.61 | 2.94 | 2.30 |
| Metabolomics ESI+ | Plasma | Phe Phe | 0.058 | 3.94E+06 | 3.14E+05 | 7.96 | 1.87 | 2.25 |
| Metabolomics ESI+ | Plasma | Palmitoyl lysophosphatidylcholine | 0.026 | 4.84E+07 | 2.19E+06 | 4.53 | 3.49 | 3.16 |
| Metabolomics ESI+ | Plasma | Cortisol | 0.040 | 6.31E+05 | 6.23E+04 | 9.87 | 4.02 | 5.39 |
| Metabolomics ESI+ | Plasma | Decanoyl-carnitine | 0.062 | 3.29E+06 | 2.48E+05 | 7.52 | 5.39 | 4.30 |
| Metabolomics ESI+ | Plasma | Hypoxanthine | 0.617 | 1.86E+07 | 1.02E+06 | 5.49 | 2.06 | 2.18 |
| Metabolomics ESI+ | Plasma | Kynurenine | 0.114 | 7.86E+05 | 8.11E+04 | 10.33 | 3.89 | 3.21 |
| Metabolomics ESI+ | Plasma | Citrulline | 0.441 | 8.53E+05 | 5.45E+04 | 6.39 | 1.66 | 1.74 |
| Metabolomics ESI+ | Plasma | Adenosine | 0.271 | 8.78E+04 | 2.77E+04 | 31.58 | 4.85 | 7.06 |
| Metabolomics ESI+ | Plasma | N-Acetyl-L-Histidine | 0.465 | 1.37E+05 | 1.99E+04 | 14.57 | 1.19 | 0.97 |
| Metabolomics ESI- | Plasma | Xanthosine | 0.141 | 1.57E+05 | 2.34E+04 | 14.90 | 0.70 | 0.53 |
| Metabolomics ESI- | Plasma | Dimethyluric acid | 0.533 | 4.70E+06 | 7.24E+05 | 15.40 | 2.79 | 1.73 |
| Metabolomics ESI- | Plasma | Theophylline | 0.067 | 4.02E+05 | 3.86E+04 | 9.59 | 1.51 | 0.88 |
| Metabolomics ESI- | Plasma | Tryptophan | 0.064 | 1.89E+07 | 1.99E+06 | 10.58 | 0.51 | 0.51 |
| Metabolomics ESI- | Plasma | Hippuric acid | 0.054 | 9.28E+06 | 1.28E+06 | 13.81 | 0.56 | 0.49 |
| Metabolomics ESI- | Plasma | *p*-cresol sulfate | 0.077 | 3.11E+07 | 2.91E+06 | 9.35 | 0.48 | 0.36 |
| Metabolomics ESI- | Plasma | *p*-cresol glucuronide | 0.061 | 7.94E+05 | 1.30E+05 | 16.44 | 0.30 | 0.33 |
| Metabolomics ESI- | Plasma | Salicyluric acid | 0.044 | 4.96E+04 | 1.03E+04 | 20.79 | 3.56 | 1.94 |
| Metabolomics ESI- | Plasma | Creatinine | 0.364 | 5.06E+04 | 9.41E+03 | 18.61 | 6.02 | 3.39 |
| Metabolomics ESI- | Plasma | Cinnamoylglycine | 0.045 | 1.95E+05 | 3.13E+04 | 16.03 | 0.33 | 0.27 |
| Metabolomics ESI- | Plasma | CMPF | 0.052 | 1.11E+07 | 1.24E+06 | 11.13 | 0.54 | 0.32 |
| Metabolomics ESI- | Plasma | Cholic acid | 0.043 | 1.38E+06 | 1.71E+05 | 12.38 | 0.29 | 0.28 |
| Metabolomics ESI- | Plasma | Indoxylsulfuric acid | 0.060 | 2.28E+07 | 1.77E+06 | 7.77 | 0.45 | 0.34 |
| Metabolomics ESI- | Plasma | Glycocholic Acid | 0.051 | 3.47E+05 | 5.32E+04 | 15.33 | 0.30 | 0.38 |
| Metabolomics ESI- | Plasma | 4-Pyridoxic acid | 0.153 | 9.15E+05 | 1.26E+05 | 13.74 | 0.75 | 0.39 |
| Metabolomics ESI- | Plasma | Indolelactic acid | 0.061 | 3.37E+06 | 5.23E+05 | 15.51 | 0.19 | 0.24 |
| Metabolomics ESI- | Plasma | Steroid(C_21_H_34_O_2_)-sulfate | 0.123 | 3.31E+05 | 2.71E+04 | 8.17 | 0.55 | 0.62 |
| Metabolomics ESI- | Plasma | Phe Phe | 0.062 | 6.64E+06 | 1.10E+06 | 16.59 | 0.26 | 0.23 |
| Metabolomics ESI- | Plasma | Kynurenine | 0.123 | 7.95E+04 | 1.15E+04 | 14.49 | 0.64 | 0.56 |
| Metabolomics ESI- | Plasma | Pantothenic Acid | 0.092 | 7.23E+05 | 1.25E+05 | 17.26 | 0.67 | 0.50 |
| Metabolomics ESI+ | Urine | Sucrose | 0.360 | 8.04E+05 | 7.16E+04 | 8.91 | 0.41 | 0.27 |
| Metabolomics ESI+ | Urine | Creatinine | 0.387 | 2.63E+07 | 3.15E+06 | 11.97 | 1.33 | 2.66 |
| Metabolomics ESI+ | Urine | Tryptophan | 0.129 | 3.39E+06 | 2.85E+05 | 8.39 | 0.88 | 0.77 |
| Metabolomics ESI+ | Urine | Hippuric acid | 0.060 | 9.01E+06 | 6.48E+05 | 7.19 | 1.37 | 0.87 |
| Metabolomics ESI+ | Urine | Adenosine | 0.243 | 1.46E+06 | 2.54E+05 | 17.35 | 1.63 | 1.21 |
| Metabolomics ESI+ | Urine | 3-Indoleacetic Acid | 0.067 | 2.11E+06 | 5.64E+05 | 26.77 | 0.89 | 0.58 |
| Metabolomics ESI+ | Urine | cAMP | 0.173 | 1.45E+06 | 1.04E+05 | 7.22 | 1.43 | 1.29 |
| Metabolomics ESI+ | Urine | Caffeine | 0.054 | 2.51E+05 | 1.28E+04 | 5.10 | 0.85 | 0.77 |
| Metabolomics ESI+ | Urine | Isobutyryl carnitine | 0.218 | 2.57E+07 | 1.97E+06 | 7.66 | 0.48 | 0.43 |
| Metabolomics ESI+ | Urine | 2-Phenylacetamide | 0.055 | 1.82E+06 | 1.27E+05 | 6.97 | 1.81 | 1.23 |
| Metabolomics ESI+ | Urine | N-Acetyl-L-Histidine | 0.429 | 5.16E+06 | 3.68E+05 | 7.13 | 0.96 | 0.60 |
| Metabolomics ESI+ | Urine | Xanthurenic acid | 0.098 | 2.91E+05 | 7.73E+04 | 26.54 | 2.05 | 1.35 |
| Metabolomics ESI+ | Urine | Tyrosine | 0.601 | 4.58E+06 | 6.33E+05 | 13.82 | 3.18 | 2.60 |
| Metabolomics ESI+ | Urine | Pantothenic Acid | 0.094 | 6.63E+06 | 6.26E+05 | 9.44 | 1.71 | 1.24 |
| Metabolomics ESI+ | Urine | Uric acid | 0.477 | 5.37E+07 | 1.36E+06 | 2.53 | 0.24 | 0.29 |
| Metabolomics ESI+ | Urine | Acetylcarnitine | 0.738 | 3.78E+07 | 5.21E+05 | 1.38 | 1.40 | 1.43 |
| Metabolomics ESI+ | Urine | 1-Methyladenosine | 1.004 | 3.48E+07 | 2.68E+06 | 7.71 | 1.87 | 1.59 |
| Metabolomics ESI+ | Urine | Niacinamide | 0.642 | 9.71E+05 | 2.01E+05 | 20.73 | 2.70 | 2.25 |
| Metabolomics ESI- | Urine | N-Acetylaspartate | 0.259 | 2.50E+07 | 1.31E+06 | 5.26 | 2.50 | 2.13 |
| Metabolomics ESI- | Urine | Uric acid | 0.352 | 7.47E+07 | 1.21E+06 | 1.62 | 1.56 | 1.25 |
| Metabolomics ESI- | Urine | Tyrosine | 0.237 | 3.24E+06 | 9.15E+04 | 2.82 | 0.24 | 0.27 |
| Metabolomics ESI- | Urine | cAMP | 0.160 | 5.12E+06 | 2.48E+05 | 4.84 | 0.25 | 0.33 |
| Metabolomics ESI- | Urine | Xanthosine | 0.110 | 5.86E+06 | 2.46E+05 | 4.20 | 0.32 | 0.32 |
| Metabolomics ESI- | Urine | Tryptophan | 0.080 | 9.16E+05 | 9.37E+04 | 10.23 | 0.63 | 0.53 |
| Metabolomics ESI- | Urine | Sinapic acid | 0.063 | 7.57E+05 | 6.94E+04 | 9.17 | 0.30 | 0.40 |
| Metabolomics ESI- | Urine | Caffeic acid | 0.078 | 3.37E+05 | 2.12E+04 | 6.28 | 0.74 | 0.60 |
| Metabolomics ESI- | Urine | Indoxylsulfuric acid | 0.129 | 5.57E+07 | 4.61E+06 | 8.27 | 0.60 | 0.56 |
| Metabolomics ESI- | Urine | Hippuric acid | 0.262 | 5.08E+07 | 1.14E+06 | 2.25 | 0.66 | 0.56 |
| Metabolomics ESI- | Urine | *p*-cresol sulfate | 0.098 | 2.40E+06 | 1.93E+05 | 8.04 | 0.30 | 0.26 |
| Metabolomics ESI- | Urine | *p*-cresol glucuronide | 0.058 | 1.13E+07 | 2.23E+06 | 19.73 | 0.50 | 0.37 |
| Metabolomics ESI- | Urine | Salicyluric acid | 0.048 | 2.88E+07 | 5.60E+05 | 1.95 | 0.37 | 0.39 |
| Metabolomics ESI- | Urine | Creatinine | 0.292 | 2.88E+06 | 1.97E+05 | 6.83 | 4.89 | 2.61 |
| Metabolomics ESI- | Urine | Ascorbic acid | 0.198 | 1.65E+06 | 6.38E+04 | 3.87 | 0.54 | 0.57 |
| Metabolomics ESI- | Urine | Cinnamoylglycine | 0.041 | 1.41E+07 | 4.43E+05 | 3.14 | 0.35 | 0.27 |
| Metabolomics ESI- | Urine | CMPF | 0.044 | 3.23E+06 | 1.72E+05 | 5.34 | 0.36 | 0.30 |
| Metabolomics ESI- | Urine | Cholic acid | 0.032 | 8.74E+05 | 6.43E+04 | 7.36 | 0.23 | 0.22 |
