# Supplementary material for: Multimodal biomarker discovery for active Onchocerca volvulus infection
Source: PLoS Negl Trop Dis. 2021 Nov 29;15(11):e0009999. doi: 10.1371/journal.pntd.0009999 (PMC8659328; doi:10.1371/journal.pntd.0009999)
Supplement: S9 Table — Precision obtained with different normalization strategies for the QC samples is shown. (DOCX) [file pntd.0009999.s013.docx]

**S9 Table.** Targeted validity verification of GC-MS based metabolomics in plasma. Precision obtained with different normalization strategies for the QC samples is shown.

| **Compound name** |  | **No Norm** | **IS Norm** | **Next QC Norm** |
| --- | --- | --- | --- | --- |
|  | **Avg** | **RSD(%)** | **RSD(%)** | **RSD(%)** |
| L-valine 1 | 275290 | 25.4 | 19.8 | 5.3 |
| L-alanine 1 | 160164 | 48.2 | 36.9 | 25.9 |
| L-proline 1 | 99717 | 17.7 | 6.5 | 5.0 |
| L-valine 2 | 146276 | 36.4 | 23.8 | 13.2 |
| urea | 2374297 | 28.0 | 16.1 | 12.5 |
| benzoic acid | 42051 | 19.3 | 9.6 | 5.6 |
| L-serine 1 | 132759 | 16.2 | 5.5 | 5.4 |
| glycerol | 363088 | 23.1 | 10.7 | 6.9 |
| L-threonine 1 | 177012 | 13.8 | 3.8 | 5.8 |
| L-proline 2 | 76489 | 21.0 | 11.3 | 12.1 |
| glycine | 718597 | 26.9 | 15.5 | 6.5 |
| succinic acid | 25363 | 16.6 | 8.4 | 6.9 |
| glyceric acid | 8145 | 18.3 | 6.6 | 6.2 |
| porphine 1 | 12997 | 40.9 | 30.0 | 18.1 |
| L-serine 2 | 61059 | 37.4 | 25.0 | 12.9 |
| L-alanine 2 | 17618 | 58.2 | 58.1 | 37.1 |
| L-threonine 2 | 28819 | 37.6 | 25.4 | 13.0 |
| L-methionine 1 | 11210 | 14.8 | 7.1 | 6.9 |
| DL-3-aminoisobutyric acid 2 | 21387 | 53.6 | 53.7 | 43.3 |
| capric acid | 4122 | 27.2 | 22.4 | 11.8 |
| iminodiacetic acid 1 | 40517 | 36.7 | 40.3 | 21.1 |
| trans-4-hydroxy-L-proline 1 | 13604 | 15.1 | 5.8 | 6.6 |
| D-threitol | 8319 | 19.8 | 7.6 | 7.0 |
| aspartic acid 2 | 17280 | 42.4 | 31.2 | 11.5 |
| L-glutamic acid 3 (dehydrated) | 465172 | 19.7 | 7.3 | 6.9 |
| L-glutamic acid 1 | 148708 | 14.0 | 8.6 | 8.7 |
| L-glutamine 1 | 128226 | 57.7 | 48.7 | 38.5 |
| Phenylalanine 1 | 142196 | 13.5 | 5.3 | 5.6 |
| alpha ketoglutaric acid | 36909 | 18.7 | 7.9 | 8.5 |
| L-glutamic acid 2 | 109250 | 51.3 | 41.3 | 26.5 |
| tartaric acid | 3220 | 14.5 | 4.7 | 8.5 |
| lauric acid | 16212 | 18.2 | 7.1 | 5.9 |
| pyrophosphate | 70201 | 76.3 | 67.6 | 53.0 |
| L-asparagine 2 | 3718 | 30.6 | 28.2 | 29.1 |
| citrulline 1 | 7010 | 33.5 | 29.6 | 22.0 |
| glycerol 1-phosphate | 89735 | 19.8 | 14.2 | 19.1 |
| hypoxanthine | 6900 | 28.3 | 18.0 | 15.0 |
| citric acid | 147510 | 18.5 | 6.5 | 6.6 |
| citrulline 2 | 2020 | 34.3 | 28.8 | 24.9 |
| Myristic Acid d27 | 7515720 | 13.5 | - | 6.8 |
| fructose 1 | 45062 | 45.8 | 42.7 | 42.1 |
| tyrosine 1 | 165205 | 17.9 | 7.5 | 10.5 |
| D-glucose 1 | 23187217 | 4.6 | 10.4 | 5.0 |
| D-glucose 2 | 4044972 | 14.0 | 3.5 | 9.6 |
| L-lysine 2 | 40010 | 34.9 | 31.8 | 35.8 |
| L-tyrosine 2 | 57341 | 31.8 | 26.0 | 28.4 |
| palmitoleic acid | 21948 | 15.9 | 5.5 | 8.3 |
| palmitic acid | 158063 | 12.7 | 11.8 | 9.6 |
| myo-inositol | 80334 | 32.3 | 22.4 | 24.7 |
| uric acid 1 | 463291 | 27.6 | 28.1 | 12.4 |
| linoleic acid | 111767 | 14.0 | 4.1 | 7.8 |
| L-tryptophan 2 | 129038 | 24.1 | 19.9 | 14.1 |
| oleic acid | 259050 | 13.1 | 5.2 | 8.1 |
| stearic acid | 227000 | 15.3 | 19.6 | 10.8 |
| alpha tocophereol | 62327 | 19.8 | 7.8 | 6.2 |
| cholesterol | 1278135 | 18.0 | 5.3 | 6.7 |
