# Supplementary material for: Multimodal biomarker discovery for active Onchocerca volvulus infection
Source: PLoS Negl Trop Dis. 2021 Nov 29;15(11):e0009999. doi: 10.1371/journal.pntd.0009999 (PMC8659328; doi:10.1371/journal.pntd.0009999)
Supplement: S10 Table — Precision obtained with different normalization strategies for the QC samples is shown. (DOCX) [file pntd.0009999.s014.docx]

**S10 Table.** Targeted validity verification of GC-MS based metabolomics in urine. Precision obtained with different normalization strategies for the QC samples is shown.

| **Compound name** |  | **No Norm** | **IS Norm** | **TMC Norm** | **TMC X IS Norm** |
| --- | --- | --- | --- | --- | --- |
|  | **Avg** | **RSD(%)** | **RSD(%)** | **RSD(%)** | **RSD(%)** |
| 2-hydroxybutyric acid | 74807 | 69.2 | 22.9 | 39.7 | 15.2 |
| glycolic acid | 248592 | 75.1 | 33.6 | 48.8 | 18.7 |
| oxalic acid | 219931 | 66.9 | 21.4 | 37.9 | 16.2 |
| p-cresol | 184267 | 74.3 | 27.3 | 42.7 | 24.9 |
| malonic acid 1 | 65858 | 79.7 | 33.3 | 51.2 | 8.9 |
| guaiacol | 8331 | 76.4 | 26.1 | 44.8 | 15.2 |
| benzoic acid | 36553 | 77.1 | 28.7 | 47.3 | 9.9 |
| phosphoric acid | 30224988 | 21.5 | 47.3 | 34.4 | 70.0 |
| glycine | 248807 | 83.0 | 48.7 | 58.8 | 49.3 |
| succinic acid | 157745 | 70.6 | 22.7 | 39.3 | 23.0 |
| catechol | 13743 | 65.8 | 15.7 | 32.3 | 26.9 |
| glyceric acid | 12295 | 65.6 | 16.3 | 33.4 | 23.7 |
| porphine 1 | 9817 | 64.7 | 29.6 | 38.3 | 35.3 |
| tartronic acid | 50405 | 64.1 | 14.1 | 32.2 | 20.9 |
| L-threonine 2 | 8830 | 87.6 | 61.5 | 71.0 | 51.2 |
| 4-methylcatechol | 6657 | 70.8 | 22.0 | 40.1 | 14.9 |
| DL-3-aminoisobutyric acid 2 | 42377 | 107.9 | 92.6 | 99.1 | 84.5 |
| citramalic acid | 18473 | 72.6 | 23.8 | 42.4 | 10.4 |
| D-malic acid | 17061 | 72.5 | 23.5 | 42.1 | 12.3 |
| D-threitol | 111554 | 60.8 | 12.5 | 30.1 | 20.3 |
| L-pyroglutamic acid | 504896 | 85.0 | 40.0 | 57.8 | 14.5 |
| Creatinine | 121485 | 102.6 | 80.0 | 88.2 | 75.6 |
| alpha ketoglutaric acid | 201215 | 51.6 | 4.2 | 19.4 | 30.2 |
| L-dithiothreitol 1 | 59189 | 79.9 | 32.9 | 51.3 | 7.5 |
| 3-hydroxyphenylacetic acid | 13384 | 72.7 | 24.8 | 43.0 | 9.4 |
| 4-hydroxyphenylacetic acid | 47616 | 69.6 | 20.0 | 38.7 | 13.7 |
| 6-deoxy-D-glucose 2 | 74563 | 79.7 | 31.5 | 50.2 | 8.1 |
| trans-aconitic acid | 151213 | 77.8 | 33.0 | 49.9 | 18.7 |
| O-phosphocolamine | 9969 | 78.2 | 36.0 | 52.1 | 16.9 |
| 10-hydroxydecanoic acid | 7474 | 73.1 | 25.7 | 43.8 | 10.4 |
| citric acid | 27818496 | 11.9 | 36.3 | 20.0 | 60.8 |
| Myristic Acid d27 | 1497509 | 51.5 | 0.0 | 18.9 | 30.5 |
| hippuric acid 2 | 4976066 | 58.7 | 8.1 | 26.7 | 24.1 |
| fructose 1 | 64785 | 69.0 | 20.5 | 38.8 | 13.3 |
| fructose 2 | 42642 | 70.5 | 22.5 | 40.6 | 13.6 |
| D-mannose 1 | 1421503 | 58.6 | 8.9 | 27.2 | 22.9 |
| D-glucose 1 | 1377551 | 54.0 | 4.0 | 21.4 | 28.6 |
| D-glucose 2 | 134790 | 70.6 | 22.0 | 40.3 | 11.6 |
| D-mannitol | 1063811 | 59.1 | 9.9 | 27.4 | 23.9 |
| galacturonic acid 2 | 95194 | 72.9 | 24.5 | 42.8 | 11.2 |
| gluconic acid 2 | 381931 | 60.0 | 11.2 | 28.9 | 22.0 |
| mucic acid | 86255 | 68.6 | 20.4 | 38.6 | 13.8 |
| xanthine | 56384 | 75.6 | 27.9 | 46.0 | 10.9 |
| N-acetyl-D-mannosamine 1 | 27256 | 83.1 | 32.0 | 50.3 | 22.9 |
| uric acid 1 | 7186864 | 31.5 | 19.8 | 7.3 | 45.9 |
| Sucrose | 264233 | 64.9 | 17.8 | 35.3 | 16.4 |
| lactose 1 | 23778188 | 5.4 | 44.0 | 28.7 | 67.1 |
| maltose 2 | 3421026 | 34.8 | 18.6 | 7.8 | 45.0 |
| lactobionic acid 2 | 78729 | 89.9 | 72.7 | 78.1 | 65.3 |
| isomaltose 1 | 67204 | 69.3 | 20.9 | 39.1 | 13.3 |
| lactobionic acid 1 | 35516 | 98.6 | 57.9 | 74.2 | 28.7 |
