# Supplementary material for: Multimodal biomarker discovery for active Onchocerca volvulus infection
Source: PLoS Negl Trop Dis. 2021 Nov 29;15(11):e0009999. doi: 10.1371/journal.pntd.0009999 (PMC8659328; doi:10.1371/journal.pntd.0009999)
Supplement: S1 Supplementary Materials and Methods — (DOCX) [file pntd.0009999.s015.docx]

**Suppl. Material and methods**

***Sample preparation***

*Sample preparation: urine for RP-LC Q-TOF-MS based metabolomics*

The frozen urine samples were thawed on ice and centrifuged at 20,627 x g for 10 min. Samples were prepared by adding 875 μL of water to 125 μL of urine in Eppendorf tubes and briefly vortex mixing, after which 100 µL was transferred to a vial with glass insert for LC-MS analysis.

*Sample preparation: urine for GC-MS based metabolomics*

The frozen urine samples were thawed on ice and 200 µL was transferred to Eppendorf tubes and 10 μL of internal standard solution (3 mg/mL myristic acid-d_27_ in water/methanol/2-propanol 2/5/2 (v/v/v)) and 150 µL of 10 mg/mL urease solution was added. Samples were placed at 37 °C for 30 min and 500 µL of ice-cold ethanol (−20 °C) was added. After vortex mixing during 30 s, samples were placed at -20 °C for 20 min. Afterwards, the samples were centrifuged for 10 min at 20,627 x g and 450 μL of the upper phase was transferred to a clear extreme recovery glass vial which was dried in a centrifugal vacuum concentrator. Next, a two-step derivatization was performed. Methoxyamination was performed by adding 100 µL of a 20 mg/mL solution of methoxyamine hydrochloride in pyridine, vortex mixing for 1 min and heating at 30°C for 90 min. An aliquot of 100 µL N-Methyl-N-(trimethylsilyl) trifluoroacetamide with 1% trimethylchlorosilane (MSTFA + 1% TMCS) was subsequently added, vortexed for 1 min and heated at 37°C for 60 min. Derivatized samples show limited stability (Fiehn and Kind, 2007), so injection was performed within 24 hours after the derivatization process.

*Sample preparation: plasma for RP-LC Q-TOF-MS based metabolomics*

The frozen plasma samples were thawed on ice. Subsequently, 50 μL of plasma was transferred to Eppendorf tubes and 200 μL of ice-cold methanol (−20 °C) was added. After vortex mixing during 30 s, samples were placed at -20 °C for 20 min. Afterwards, the samples were centrifuged for 10 min at 20,627 x g and 200 μL of the supernatant was dried in a centrifugal vacuum concentrator. The dried extracts were dissolved in 100 μL of water/acetonitrile 95/5 (v/v) and transferred to a new Eppendorf tube. The samples were centrifuged for 10 min at 20,627 x g, after which 80 μL of supernatant was transferred to a vial with glass insert for LC-MS analysis.

*Sample preparation: plasma for GC-MS based metabolomics*

The frozen plasma samples were thawed on ice and 50 µL was transferred to Eppendorf tubes and 10 μL of internal standard solution (3 mg/mL myristic acid-d_27_ in water/methanol/2-propanol 2/5/2 (v/v/v)) and 500 µL of ice-cold methanol (−20 °C) was added. After vortex mixing during 30 s, samples were placed at -20 °C for 20 min. Afterwards, the samples were centrifuged for 10 min at 20,627 x g and 450 μL of the upper phase was transferred to a clear extreme recovery glass vial which was dried in a centrifugal vacuum concentrator. Next, a two-step derivatization was performed as described for the urine samples.

*Sample preparation: plasma for RP-LC Q-TOF-MS based lipidomics*

The frozen plasma samples were thawed on ice. Subsequently, 50 μL of plasma was transferred to Eppendorf tubes and 300 μL of methanol was added. After vortex mixing during 10 s, 1,000 µL of *tert*-butyl methyl ether was added and samples were incubated at room temperature for 1 hr in a shaker. Afterwards, 260 µL of water was added and samples were incubated at room temperature for 10 min in a shaker. The samples were centrifuged for 10 min at 1,000 x g and 900 μL of the upper phase was transferred to a high-recovery vial which was dried in a centrifugal vacuum concentrator. The dried extracts were dissolved in 90 µL isopropanol / methyl tert-butyl ether 50/50 (v/v).

***RP-LC Q-TOF-MS based lipidomics***

The LC-MS method was adapted from Sandra *et al.* and T’Kindt *et al.* (1, 2). The samples (10-µL injection volume) were chromatographically separated on an Acquity UPLC BEH Shield RP18 column (2.1 x 100 mm; 1.7 μm; Waters, Milford, MA, USA). Chromatographic separation was achieved on an Agilent 1290 Infinity LC system (Infinity Binary Pump G4220A, Thermostat G1330B, Infinity Sampler G4226A; Agilent Technologies). The column temperature was maintained at 80°C using a stand-alone Sandra/Selerity Series 9000 Polaratherm oven (Selerity Technologies, Salt Lake City, UT, USA). Eluting buffers were buffer A (20 mM ammonium formate, pH 5) and buffer B (MeOH). Starting conditions were 50 % buffer B at a flow rate of 0.5 mL/min. Over 5 min a gradient was applied to 74 % B. Thereafter the percentage of buffer B was increased to 100% in subsequent gradients (5-6 min 74-85% B; 6-16 min 85-90% B; 16-17 min 90-94% B; 17-26 min 94-100% B), before returning to the starting conditions (50% B) which was hold for 9 min.

High-resolution accurate mass spectra and fragmentation spectra were obtained with an Agilent 6550 Q-TOF mass spectrometer (MS) (Agilent Technologies) equipped with Dual Jetstream electrospray ionization (ESI) source. The instrument was operated in both positive and negative electrospray ionization mode. Needle voltage was optimized to +/- 3.5 kV, the drying and sheath gas temperatures were set to 290°C and 400°C and the drying and sheath gas flow rates were set to 13 and 12 L/min, respectively. Data were collected in centroid mode from *m/z* 100–1700 in positive ion mode and *m/z* 100-1700 in negative ion mode at an acquisition rate of 2 spectra/s in the extended dynamic range mode (2 GHz), offering an in-spectrum dynamic range of 10^5^ and a resolution of ± 20000 FWHM in the lipid *m/z* range. To maintain mass accuracy during the analysis sequence, a reference mass solution was used containing reference ions (*m/z* 121.050873 and 922.009798 for positive ESI mode, *m/z* 112.985587 and 1033.988109 for negative ESI mode). MS/MS experiments were performed in the targeted MS/MS mode, thereby adding precursors of interest in an inclusion list. The quadrupole was operated at narrow resolution (1.3 amu window) and the collision energy was fixed at either 20 or 35 eV. Data acquisition was performed using MassHunter Acquisition B.06.01.

***RP-LC Q-TOF-MS based metabolomics***

The LC-MS method was adapted from Boelaert *et al.* (3). The samples (2-µL injection volume) were chromatographically separated on an Acquity UPLC HSS T3 column (2.1 x 100 mm; 1.8 μm; Waters, Milford, MA, USA). Chromatographic separation was achieved on an Agilent 1290 Infinity LC system (Infinity Binary Pump G4220A, Thermostat G1330B, Infinity Sampler G4226A, Infinity Thermostatted Column Compartment G1316C; Agilent Technologies). The column temperature was maintained at 40°C. Eluting buffers were buffer A (0.1% HCOOH in H2O) and buffer B (0.1% HCOOH in acetonitrile). Starting conditions with 0 % buffer B at a flow rate of 0.35 mL/min was held for 1 min. Subsequently, over 15 min a gradient was applied to 100% B followed by an isocratic hold for 4 min before returning to the starting conditions (0% B) for 5 min.

High-resolution accurate mass spectra and fragmentation spectra were obtained with an Agilent 6550 Q-TOF mass spectrometer (MS) (Agilent Technologies) equipped with a Dual Jetstream electrospray ionization (ESI) source. The instrument was operated in both positive and negative electrospray ionization mode. Needle voltage was optimized to +/- 3.5 kV, the drying and sheath gas temperatures were set to 290°C and 400°C and the drying and sheath gas flow rates were set to 13 and 12 L/min, respectively. Data were collected in centroid mode from *m/z* 100–1700 in positive ion mode and *m/z* 100-1700 in negative ion mode at an acquisition rate of 2 spectra/s in the extended dynamic range mode (2 GHz), offering an in-spectrum dynamic range of 105 and a resolution of ± 10000 FWHM in the lipid *m/z* range. To maintain mass accuracy during the analysis sequence, a reference mass solution was used containing reference ions (*m/z* 121.050873 and 922.009798 for positive ESI mode, *m/z* 112.985587 and 1033.988109 for negative ESI mode). MS/MS experiments were performed in the targeted MS/MS mode, thereby adding precursors of interest in an inclusion list. The quadrupole was operated at narrow resolution (1.3 amu window) and the collision energy was fixed at either 20 or 35 eV. Data acquisition was performed using MassHunter Acquisition B.06.01.

Data analysis was performed using the MassHunter Workstation Software, Profinder, and the Mass Profiler Professional (MPP) Software: MassHunter Qualititative Analysis (Version B.07.00 Build 7.0.7024.29, Service Pack 1), Profinder (Version B.06.00), and MPP (Version 12.1 Build 170166). Raw LC-MS data files were processed in an untargeted fashion using the Molecular Feature Extraction (MFE) algorithm incorporated in the MassHunter Profinder software package. This feature extraction algorithm localizes the unique peaks in the LC-MS chromatogram. Each feature is composed of a retention time, mass and intensity. For comparative metabolomics or lipidomics, the resulting feature files from MFE were imported in MassProfiler Professional 12.0 (Agilent Technologies) which aligned, visualized and filtered the features. For statistical analysis (Mann-Whitney-U test), no filters or normalization steps were performed. Zero values were excluded from the calculation of p-values and fold changes. No multiple testing correction was executed for the statistical analysis, implying the presence of many false positives in the resulting feature lists.

In order to uncover markers for Onchocerciasis, the following strategy was used: i) Features should be highly upregulated or uniquely present (FC > 16) in nodule positive samples compared to non-endemic controls; ii) Features can be highly upregulated or uniquely present (FC > 16) in both nodule positive and lymphatic filariasis samples compared to non-endemic controls. This may incorporate markers related to (worm) infection; and iii) Features can be upregulated in nodule positive versus lymphatic filariasis samples. Here, the feature intensity should be at least two-fold higher or lower (|FC| >= 2) in nodule positive samples compared to lymphatic filariasis samples, to be able to make a distinction between these study groups.

Next to this approach, a small cohort approach was also included. A small cohort of samples from each study group (typically 8 nodule positive, 8 LF infected, 8 non-endemic controls and 4 quality control samples) was used in the data processing workflow. This workflow enabled the extraction of lower intense features (feature peak height > 2500 counts), but induced lots of false positive hits. Relevant features were extracted in the full cohort and their statistical relevance was calculated. The results of the small cohort approach are included in the resulting tables.

Statistically significant features (p-value < 0.05) containing the appropriate fold change differences were exported and once again extracted from the raw data, a process named recursion. Here, all the extracted ion chromatograms were manually checked and false positives were excluded. All compounds checked in the recursion step were again subjected to the Mann-Whitney-U test. In these analyses correction for multiple testing was performed with the Benjamini Hochberg false discovery rate.

Metabolite or lipid identification results from an identification strategy that fully exploits the features of the Q-TOF MS system. Generation of molecular formulas, based on accurate mass, isotope abundance, and isotope spacing both in positive and negative ionization mode, was complemented with accurate mass database searching the Metlin spectral library (Agilent Technologies) and an in-house build lipid database (populated with LIPID MAPS entries and theoretical lipid structures) and MS/MS measurements in both ionization modes. For lipids, fragmentation mechanisms/spectra in both positive and negative ionization modes have extensively been reported in literature (1). Each lipid class displays a characteristic fragmentation pattern in positive and/or negative ESI mode, through neutral loss or the presence of unique fragment ions. Other parameters such as lipid elution behavior and adduct formation interpretation further assisted in the identification.

For metabolites, MS/MS spectra were matched on the Metlin spectral library (Agilent Technologies), compared with literature or interpreted using MassHunter Molecular Structure Correlator (MSC; Agilent Technologies). MSC tries to explain each observed fragment ion into the proposed structure using a “systematic bond-breaking” approach as described by Hill and Mortishire-Smith (4). Molecular structures from Metlin, Human Metabolome Database (HMDB) and ChemSpider were imported into MSC, which scores the observed fragment ions from 0-100 to the theoretical fragmentation of the imported structure.

***GC-MS based metabolomics***

The GC-MS method was adapted from Fiehn and Kind (5). The samples (1-µL injection volume) were chromatographically separated on a DuraGuard DB-5MS capillary column (30 m × 0.25 mm × 0.25 μm with a 10 m guard capillary; Agilent Technologies). Chromatographic separation was achieved on an Agilent 7890A GC system (GC oven G3440A, injector G4513A, autosampler 7693; Agilent Technologies). Injection temperature was 250°C, carrier gas was Helium and injection mode was splitless. Flow was constant at approx.35 cm/s and following temperature was employed: 60°C for 1 min, 10°C/min to 325°C and 3525°C for 10 min.

Data acquisition was performed using MassHunter GC/MS Acquisition B.07.04.2260. Mass spectra were obtained with an Agilent 5975 inert XLMass Selective Detector (G3174A; Agilent Technologies).

Retention time was locked to myristic-d27 acid at 16.727 min. These conditions are based on the library developed by Kind et al. [4]. Electron ionization (EI) was used and MS was performed in scan mode (*m/z* 50-600) with the MS quadrupole at 150°C and MS ion source at 250°C. The system was tuned using perfluorotributylamine (PFTBA). The GC-MS analytical block has been divided into six analytical batches with 15 to 17 study samples per batch, 5 conditioning samples (matrix samples) and 4 QC samples.

Data analysis was performed using the MassHunter Workstation Software: MassHunter Qualititative Analysis (Version B.07.00 Build 7.0.7024.0), MassHunter Quantitative Analysis (Version B.07.01 Build 7.1.524.2, Service Pack 2) and MPP (Version 12.1 Build 170166). Raw GC-MS data files were processed in an untargeted fashion using the Find Compounds by Chromatogram Deconvolution algorithm in the MassHunter Qualitative and Quantitative software packages. This algorithm extracts the deconvoluted spectrum for each peak in the GC-MS chromatogram. Each compound is composed of a retention time, a mass (most abundant or specific mass of the deconvoluted mass spectrum), intensity of this mass and the compound spectrum. All integrations were manually checked using MassHunter Quantitative software for all peaks in all samples.

For comparative metabolomics, the resulting compound lists from MassHunter Quantitative software were imported in Excel (Version 14.0.7177.5000) (Microsoft Office Home and Business 2010) and different calculations were applied, especially regarding data normalization. The resulting data matrices were then imported in MPP which aligned, visualized and filtered the features. Differences in metabolite expression between batches were calculated using Mann-Whitney-U test with Benjamini Hochberg false discovery rate correction.

Identification of the selected features was performed by matching the mass spectrum and the retention time of the feature with the Fiehn library. When not found, the mass spectrum was matched with the NIST11 library.

***RP-LC Q-TOF based targeted lipid analysis***

The samples (10 µL injection volume) were chromatographically separated on an Acquity UPLC HSS T3 column (2.1 x 100 mm; 1.8 μm; Waters, Milford, MA, USA). Chromatographic separation was achieved on an Agilent 1290 Infinity UHPLC system (Infinity High Speed Pump G7120A, Thermostated column department G7116B, Infinity Sampler G7167B; Agilent Technologies). The column temperature was maintained at 60°C. Eluting buffers were buffer A (10 mM ammonium formate, 0.1% formic acid in H_2_O) and buffer B (10 mM ammonium formate, 0.1% formic acid in isopropanol/acetonitrile 90/10 (v/v)). Starting conditions were 30 % buffer B at a flow rate of 0.4 mL/min. For 1 min this starting condition was maintained. Thereafter the percentage of buffer B was first increased to 100% in subsequent gradients (1-3 min 30-70% B; 3-8 min 70-80% B; 8-8.1 min 80-100% B), where it was maintained for 5 min (8.1-13 min), before returning to the starting conditions (30% B) which was hold for 5 min.

High-resolution accurate mass spectra were obtained with an Agilent Q-TOF 6545 mass spectrometer (MS) (Agilent Technologies) equipped with Dual Jetstream electrospray ionization (ESI) source. The instrument was operated in negative electrospray ionization mode. Needle voltage was optimized to +/- 3.5 kV, the drying and sheath gas temperatures were set to 300°C and 350°C and the drying and sheath gas flow rates were both set to 8 L/min. Data were collected in centroid mode from *m/z* 100–1700 in negative ion mode at an acquisition rate of 2 spectra/s in the extended dynamic range mode (2 GHz). To maintain mass accuracy during the analysis sequence, a reference mass solution was used containing reference ions (*m/z* 112.985587 and 1033.988109). Data acquisition was performed using MassHunter Acquisition B.06.01.

***RP-LC MS/MS based targeted metabolite analysis***

The samples (2-µL injection volume) were chromatographically separated on an Acquity UPLC HSS T3 column (2.1 x 100 mm; 1.8 μm; Waters, Milford, MA, USA). Chromatographic separation was achieved on an Acquity UPLC system (Waters, Milford, MA, USA) equipped with a binary solvent manager, sample manager, sample organizer and column oven. The column temperature was maintained at 50°C. Eluting buffers were buffer A (0.1% formic acid in H_2_O) and buffer B (acetonitrile). Starting conditions were 5 % buffer B at a flow rate of 0.5 mL/min. Thereafter, the percentage of buffer B was increased to 80% in a gradient over 3min. Subsequently, the percentage of buffer B was increased to 90% where it was maintained for 1 minute before returning to the starting conditions (5 % B) which was held for 1 min.

High-resolution accurate mass spectra and fragmentation spectra were obtained with a TripleTOF 6600 Q-TOF mass spectrometer (AB Sciex, Ontario, Canada) equipped with a Turbo V electrospray ionization (ESI) source. The instrument was operated in electrospray ionization in the negative ion mode. Ion spray voltage was set to - 4.5 kV, source temperature was set to 550ºC. The nebulizer gas was set to 50 psi and the heater gas to 55 psi, the de-clustering potential to -80 V.

Data were collected in both untargeted TOFMS (*m/z* 100–2000, collision energy 10V, accumulation time 100 ms) and targeted TOFMS/MS (collision energy -30V with a spread of 15V, accumulation time 80 ms) experiments. During MS/MS experiments precursors of interest were included in an inclusion list. The instrument operated at high resolution mode (~30 000 FWHM) for TOFMS scans and high sensitivity mode (~15 000 FWHM) for TOFMS/MS scans. Mass calibrations were performed using positive ESI calibration solution (AB Sciex), which was automatically delivered by a calibrant delivery system (AB Sciex). Data acquisition was performed using the Analyst TF 1.7.1 software (AB Sciex). Quantification data were obtained using the Multiquant 3.0.2 software. Individual compounds were quantified using the peak area’s of the selected fragment ions.

***Synthesis of cis-cinnamoylglycine***

Starting material H-Gly-2-chlorotrityl resin (or glycine 2-chlorotrityl ester polymer-bound, ~1.1 mmol/g loading), HATU, and *N*-(trans-cinnamoyl)glycine (CAS# 16534-24-0) were purchased from Sigma-Aldrich (Saint Louis, MO, USA). cis-Cinnamic acid was purchased from DSK Biopharma, Inc. (Morrisville, NC, USA). Other reagents and solvents were obtained from VWR International.

^1^H and ^13^C NMR spectra were acquired on a Bruker 300-Avance (300 MHz) spectrometer with TMS as an internal standard. Chemical shifts were expressed in parts per million (ppm,
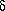
scale). Low-resolution LCMS analysis was performed on an Agilent 1200 series HPLC/ AB Sciex API4000 mass spectrometer with an Agilent Zorbax SB C18 column (3 μm, 2.1 × 50 mm), and with gradient elution from 10-100% CH_3_CN-H_2_O containing either 0.05% TFA or 0.05% NH_4_OAc over 3.5 min, then held at 100% CH_3_CN for 2.5 min. The flow rate was 0.5 mL/min, UV detection at 214 and 254 nm, mass scan range was 120-1500 amu. High-resolution LCMS analysis was performed on an Agilent 1200 series HPLC and 6230 TOF mass spectrometer. Flash chromatography was performed using a Teledyne Isco CombiFlash Companion system and a RediSep silica gel column unless otherwise specified. Reverse-phase preparative HPLC purifications were performed using a Gilson system equipped with a Phenomex Kinetex XB-18 column (5 um, PN 00G-4605-P0-AX, SN H15-091816, BN 5705-0037).

The H-Gly-2-Chlorotrityl resin (0.5 g, ~1.1 mmol/g loading) was suspended in 3 ml of THF, Et_3_N was added, and then cis-cinnamic acid (74.1 mg) and HATU (209.1 mg) in such sequence were added. The suspension was stirred at room temperature overnight. The suspension was then filtered and rinsed with THF (5 x 5 mL). And the resin was further rinsed with water (7 x 7 mL) and then THF (2 x 5 ml). The remaining resin was then washed with 0.5% TFA dichloromethane (10 x 6 mL) (the resin changed to deep red colored) to give a colorless solution. The solution was evaporated to dryness to give a crude product as a colorless oil (152 mg with some THF residue).

Column chromatography on a 12-g silica gel with 0-20% methanol/DCM gave the product (84 mg) in 92+% UV purity. Column chromatography of this product on a 12-g Hilic cartridge with 0-10% methanol/DCM gave the product in 97+% UV purity (45 mg) and other less pure fraction 17 mg. The latter was further purified Gilson HPLC with the Phenomex Kinetex XB-18 column, injection 100-200 ul methanol solution, 3 times, flow rate 20 ml/min, 10-90% ACN/water with 0.1% TFA from 0-20 min, run time ~30 min, UV 254 and 215 nm, Tr ~10 min. The collected fractions were combined and evaporated to dryness, dissolved in methanol and re-evaporated to dryness again. This product was obtained as colorless film (12.0 mg).

This product was found to have an UV chromatographic purity of 98+% by area normalization of the detected peaks at a wavelength of 254 nm on an Agilent 1100 series HPLC analysis with a Waters XBridge C18 5um 250X4.6mm column; Mobile phase: A: 0.1% TFA in water, B: 0.1% TFA in CH3CN; gradient from 10%B to 20%B in 0-20 min, gradient from 20%B to 90%B in 20-30 min, isocratic at 90%B in 30-33 min, gradient from 90%B to 10%B in 33 to 33.1 min, isocratic at 10%B in 33.1-40 min, post run 3 min; flow rate 1 mL/min, injection volume 5 uL of methanol solution at 1 mg/ml; Column temperature 25°C. Retention time Tr = 18.3 min.

HNMR in deuterated methanol and LCMS confirmed the product which was different to authentic *N*-(trans-cinnamoyl)glycine on HPLC analysis. LC-MS analysis (Agilent 1200 HPLC series/ AB Sciex API4000) showed LR-MS, m/z 206 (M+H^+^), HR-TOFMS (Agilent 1200 HPLC series/ 6230 TOF LCMS) m/z calcd for M+H^+^ (C_11_ H_12_ NO_3_) 206.081, found 206.082. Proton coupling constant of the vicinal olefin of the product was found to be 12.6 Hz in deuterated methanol while N-(trans-cinnamoyl)glycine had the coupling constant as 16.1 Hz. This showed the obtained product had the cis-configuration. ^1^H NMR (CD_3_OD) δ 7.56-7.54 (m, 2H), 7.32-7.28 (m, 3H), 6.78 (d, *J* = 12.6 Hz, 1H), 6.05 (d, *J* = 12.6 Hz, 1H), 3.94 (s, 2H). ^13^C NMR (CD_3_OD) δ 173.1 (*C*O), and 170.1 (*C*O), 138.8 (*C*H), 136.4 (t-*C*), 130.7 (*C*H), 129.6 (*C*H), 129.2 (*C*H), 123.7 (*C*H), 41.9 (*C*H_2_).

**Supplementary References**

1. Sandra K, Pereira Ados S, Vanhoenacker G, David F, & Sandra P (2010) Comprehensive blood plasma lipidomics by liquid chromatography/quadrupole time-of-flight mass spectrometry. *Journal of chromatography. A* 1217(25):4087-4099.

2. t'Kindt R*, et al.* (2015) Profiling over 1500 lipids in induced lung sputum and the implications in studying lung diseases. *Analytical chemistry* 87(9):4957-4964.

3. Boelaert J*, et al.* (2014) State-of-the-art non-targeted metabolomics in the study of chronic kidney disease. *Metabolomics* 10(3):425-442.

4. Hill AW & Mortishire-Smith RJ (2005) Automated assignment of high-resolution collisionally activated dissociation mass spectra using a systematic bond disconnection approach. *Rapid Communications in Mass Spectrometry* 19(21):3111-3118.

5. Fiehn O & Kind T (2007) Metabolite profiling in blood plasma. *Methods in molecular biology* 358:3-17.
